# From evidence to action: Italian recommendations for the diagnosis and treatment of spatial neglect in stroke patients

**DOI:** 10.1007/s10072-026-09115-z

**Published:** 2026-05-28

**Authors:** Mauro Mancuso, Simona Vecchi, Benedetta Basagni, Michele Basile, Gabriella Bottini, Paolo Caffarra, Fabio Cruciani, Gaspare Galati, Cecilia Guariglia, Luisa Magnotti, Suzanna Mitrova, Marianna Purgato, Nicoletta Reale, Cristina Reverberi, Rosella Saulle, Matteo Sozzi, Valentina Varalta, Ilaria Valentini, Pierluigi Zoccolotti

**Affiliations:** 1 Physical and Rehabilitative Medicine Unit, Az. USL South-East Tuscany, Grosseto, Italy; 2 Fondazione Gianfranco Salvini ETS, Montevarchi (AR), Italy; 3 Department of Epidemiology, Lazio Regional Health Service - ASL ROMA, Rome, Italy; 4 Tuscany Rehabilitation Clinic, Piazza del Volontariato, 1, Montevarchi, Arezzo, Italy; 5 Graduate School of Health Economics and Management (ALTEMS), Catholic University of Sacred Heart, Rome, Italy; 6 Department of Brain and Behavioral Sciences, University of Pavia, Pavia, Italy; 7 National Committee on Dementia, Italian Ministry of Health, Rome, Italy; 8 Department of Psychology, Sapienza University of Rome, Rome, Italy; 9 IRCCS Santa Lucia Foundation, Rome, Italy; 10 Department of Neuroscience, Biomedicine and Movement, University of Verona, Verona, Italy; 11 A.L.I.Ce. Italia Odv, Rome, Italy; 12 Az. USL Reggio Emilia, Reggio Emilia, Italy; 13 Casa Maria delle Grazie, Nibionno, Lecco, Italy

**Keywords:** Spatial neglect, Stroke rehabilitation, Prism adaptation therapy, Visuospatial training, Clinical practice guidelines

## Abstract

**Background:**

Spatial neglect is a common and disabling cognitive consequence of stroke, associated with reduced functional independence, prolonged hospitalization, and increased healthcare and societal burden.

**Objective:**

The aim of this paper is to describe the development of a Clinical Practice Guideline (CPG) for the diagnosis and treatment of spatial neglect in stroke patients.

**Methods:**

The guideline was developed by an interdisciplinary expert Panel following the GRADE approach, supported by systematic reviews of the literature. Four clinical questions addressed the effectiveness of prism adaptation therapy (PAT) and visuospatial training (VST), optimal timing of treatment initiation, and the accuracy of neglect-specific versus generic ADL assessment tools.

**Results:**

Evidence from randomized controlled trials indicates that both PAT and VST yield small, short-term improvements in ADL performance and neglect severity; however, overall certainty of evidence remains very low due to methodological limitations. Based on this evidence, the Panel developed two conditional recommendations in favor of using PAT and VST for the rehabilitation of patients with peri-personal neglect. No studies examined optimal timing, leading the Panel to issue Good Practice Statements recommending initiation within 4–7 days post-stroke. Observational evidence supports the use of neglect-specific tools, particularly the Catherine Bergego Scale (KF-NAP version) and the semi-structured Zoccolotti scale, which outperform generic ADL measures.

**Conclusions:**

This CPG represents the first coordinated national effort in Italy to standardize the diagnosis and rehabilitation of post-stroke spatial neglect within the National Health Service. The CPG provides two conditional recommendations for treatments along with practical indications to support implementation and future research.

**Supplementary Information:**

The online version contains supplementary material available at 10.1007/s10072-026-09115-z.

## Introduction

### Definition, epidemiology, and classification

Spatial neglect is a multifaceted neuropsychological syndrome characterized by a failure to attend, respond, or orient to stimuli presented in the contralesional hemispace. The disorder can manifest across multiple functional domains, including sensory–perceptual, motor–intentional, and representational (imaginal) components,and may affect different spatial sectors, namely personal, peri-personal, and extra-personal space [1]. Furthermore, spatial neglect can be dissociated according to the spatial reference frame involved, presenting in either an egocentric (viewer-centered) or an allocentric (object-centered) coordinate system.

The prevalence of the disorder is estimated to be around 29% of patients with unilateral brain lesions. It is more frequent in patients with right-hemisphere damage compared to those with left-hemisphere damage (38% vs. 18%), and in acute patients (within the first week: 45%) compared to those in the subacute phase (from 1 week to 3 months: 40%), post-acute phase (from 6 months to 1 year: 29%), or chronic phase (beyond 1 year: 20%) [1].

The incidence of this disorder ranges, depending on the study, from 12% to 95% of patients with right-hemisphere damage due to stroke (see Robertson & Halligan, 1999 [2]). This wide variability largely reflects the lack of unified, sensitive assessment criteria capable of identifying and capturing the heterogeneous features that constitute the syndrome [3].

### Clinical presentation

Neglect significantly limits the recovery of independence in activities of daily living (ADL). For instance, it affects the ability to explore the surrounding (extra-personal) space during mobility, causing a higher incidence of accidents and an increased risk of falls. Limited exploration of peri-personal space further impairs the execution of ecological routine daily activities such as eating, reading, using mobile phones and computers. Personal-space neglect impacts self-care tasks (such as grooming, washing, and dressing).

Patients with neglect and left hemiplegia exhibit poorer motor recovery compared to patients with no neglect as well as patients with right hemiplegia [4]. They also tend to have longer lengths of stay in rehabilitation facilities [5–8] and fail to achieve a level of functional recovery sufficient for safe discharge at home [6, 7]. Recovery is delayed also because neglect is also frequently associated with anosognosia, that is, poor awareness of deficits that remain unrecognized despite their obviousness [9]. Patients with anosognosia fail to understand their difficulties in performing daily activities, sometimes providing implausible explanations for their failures [10, 11]. Anosognosia reduces the patient level of collaboration, with a low an adherence to the rehabilitation plan (Paolucci et al., 2012 [12]), prolonging recovery time, and reducing gains from rehabilitation [13].

Neglect is a multifaceted syndrome, including clinical dissociations, requiring an extensive and comprehensive assessment as a prerequisite for developing individualized rehabilitation plans that consider each patient’s unique characteristics. A recent international survey [8] involving 33 countries revealed substantial variability and a lack of consensus regarding diagnostic tools and rehabilitation approaches.

### Aim of the guideline

Despite the well-established clinical relevance of post-stroke spatial neglect, Clinical Practice Guidelines (CPG) addressing its diagnosis and rehabilitation were not available within the Italian healthcare context at the time this multi-society initiative was launched. Given the complexity of the topic, the working group prioritized a limited number of clinically and organizationally relevant key questions in the development of this first national CPG. These include questions related to the efficacy and safety of the available rehabilitation treatments, such as prism adaptation therapy (PAT) and visuospatial training (VST), the appropriate timing for rehabilitation, and the accuracy of specific assessment tools for measuring ADLs in people affected by stroke-related neglect. This paper intends to illustrate the development of the CPG.

Given the complexity of the topic, the working group prioritized a limited number of clinically and organizationally relevant key questions in the development of this first national CPG.

## Methods

The development process of the CPG followed the *Methodological Manual for the Production of Clinical Practice Guidelines* [14] of the National Centre for Clinical Excellence, Healthcare Quality and Patient Safety (CNEC). We adopted the GRADE (Grading of Recommendations Assessment, Development and Evaluation) methodology for evaluating the evidence and formulating clinical recommendations. During the drafting of the CPG, we followed the AGREE Reporting Checklist [15].

### Selection of clinical questions

A multidisciplinary Panel was formed, comprising 17 professionals (including neurologists, neuropsychologists, nurses, occupational therapists, physiatrists, psychologists, and speech therapists) from fourteen scientific and volunteer societies (see Acknowledgements) to provide clinical guidelines to be shared with the Istituto Superiore di Sanità (ISS; Italian National Institute for Health). The Panel developed the following questions based on the PICO (Population, Intervention, Comparison, Outcome) framework and established explicit inclusion and exclusion criteria for study selection (see Appendix for details).

- PICO 1: In patients with unilateral spatial neglect after stroke, is prism adaptation therapy more effective than other interventions for improving functional outcomes?
- PICO 2: In patients with unilateral spatial neglect after stroke, should visuospatial training be used compared with other interventions for improving functional outcomes?
- PICO 3: In patients with peri-personal neglect after stroke who are candidates for rehabilitation, when should prism adaptation or visuospatial training be initiated to optimize functional outcomes?
- PICO 4: In stroke survivors with peri-personal neglect, are neglect-specific scales (e.g., the CBS–KF-NAP, the Zoccolotti semi-structured scale) more accurate than non-specific ADL measures (e.g., Barthel Index, FIM) for evaluating functional status?

For each PICO, a list of outcomes was selected by ranking and consensus and the following outcomes were prioritized using GRADE formal processes: critical outcomes such as functional improvement, functional independence, improvement on neuropsychological tests, length of hospital stay, quality of life, important outcomes such as mood, number of falls, and discharge destination.

### Literature review and assessment of quality of evidence

For each clinical question, a systematic review of the literature was carried out in accordance with the Preferred Reporting Items for Systematic Reviews and Meta-Analyses (PRISMA) checklist [16]. The review protocols are available in the Supplementary Materials.

To identify eligible studies, we conducted comprehensive searches up to January 2023 across the following biomedical databases: Cochrane Library Medline, Embase, PsycInfo, CINAHL, and Web of Science. Randomized controlled trials (RCTs) were used as the primary source of evidence. Relevant RCTs were identified from systematic reviews if available, followed by additional searches to identify more recently published RCTs. When these were not available, we included observational studies relevant to the clinical question of functional assessment. Search strategies were tailored for each database, combining MeSH terms with free-text keywords to optimize results. The full search strategies for each clinical question are provided in the Supplementary Materials.

Two reviewers independently screened titles and abstracts, followed by an assessment of full texts for eligibility. We evaluated the methodological quality of all included studies, applying the AMSTAR 2 [17] to systematic reviews and the Cochrane risk-of-bias [18] to randomized controlled trials.

Where possible, the study findings were meta-analyzed in Review Manager (RevMan 5.4). For outcomes, the Panel prioritized continuous measures. Effect estimates were calculated as standardized mean differences (SMDs) with 95% confidence intervals. Meta-analyses used either post-intervention or follow-up mean scores and applied a fixed-effect model. Statistical heterogeneity was assessed. For systematic reviews that already reported pooled effect sizes, combined estimates were extracted directly from these studies. In instances where quantitative synthesis was not feasible, the findings were summarized in a narrative format.

For critical outcomes, including treatment efficacy, improvement in ADL performance, and reduction of neglect symptoms, the overall certainty of evidence was assessed using the GRADE approach [19, 20]. A concise overview of these assessments is provided in the Summary of Findings tables, with detailed GRADE Evidence Profiles available for each clinical question in the Supplementary Materials. Using the GRADE approach, the overall quality of evidence for each outcome can be rated into one of four categories, as reported in Table 1.

**Table 1:** Grading/classification of the quality of evidence

Quality of evidence level	Meaning	Consequence
High	High level of confidence in the results	It is very unlikely that further studies will substantially change the current level of confidence in the effect estimate.
Moderate	Moderate level of confidence	Further studies are likely to confirm or modify confidence in the effect estimate.
Low	Results are not very reliable	Additional studies are needed to obtain reliable estimates of the intervention’s benefits and harms.
Very low	Data examined are completely unreliable	They cannot be trusted. Any effect estimate is highly uncertain.

### From evidence to recommendations

Based on the identified studies and the results of the evidence quality assessment, the Panel defined both the direction and the strength of each recommendation for each clinical question. For Clinical Questions 1 and 2, the decision-making process was supported by the Evidence to Decision (EtD) framework [21], which considers the following domains: desirable and undesirable effects of the intervention, balance of benefits and harms, confidence in patient values and preferences, resource use, equity, acceptability and feasibility of the intervention. All these elements contribute to determining the direction and strength of the recommendation. The EtD tables for clinical Questions 1 and 2 are available in the Supplementary Materials of the guideline.

The strength of a recommendation reflects the degree of certainty that the desirable effects of an intervention outweigh the undesirable ones. According to the GRADE approach, the strength of a recommendation can be classified as either strong or conditional, each associated with a level of uncertainty and specific implications (Table 2).

**Table 2:** Implications of strength of recommendation in the GRADE system

Strength	Patients	Clinicians	Policymakers
Strong	Almost all properly informed patients should follow the recommendation whereas only a small fraction of them may choose different options.	Most patients should receive the recommended intervention.	The recommendation can serve as a basis for planning and allocating available resources.
Conditional	The majority of properly informed patients will follow the recommendation, but a minority of them may choose different options.	The final choice should include a careful consideration of patients’ values and preferences.	Stakeholders should engage in discussions to address the issue before implementing the recommendation.

In specific circumstances, the Panel expressed recommendations for future research. For clinical questions in which the Panel had high confidence that the desirable effects of an intervention outweigh its undesirable effects, but the supporting evidence was indirect, the Panel issued Good Clinical Practice (GCP) statements. These statements consist of operational messages, considered relevant and necessary for clinical practice, although not suitable for formal GRADE assessment.

### External review

A multidisciplinary Panel of experts critically appraised the draft guideline, providing overall judgments, comments, and recommendations to refine the document. The Panel examined all feedback and integrated the suggested revisions into the final version of the text.

## Results

For Clinical Questions 1 and 2, the Panel formulated formal recommendations using the GRADE approach which is based on a systematic assessment of the certainty of evidence. In contrast, for Clinical Questions 3 and 4, such evidence-based recommendations could not be made, as no direct comparative studies among the available therapeutic options were identified. Instead, the Panel developed indications for good clinical practice, following the methodological manual of the National Guideline System. These indications serve as operational guidance on clinical aspects lacking comparative evidence. Unlike GRADE recommendations, these are grounded in the collective clinical experience and unanimous agreement of the panelists, not on formal evidence evaluations.

Therefore, the indications for Clinical Questions 3 and 4 should be considered pragmatic clinical guidance, reflecting expert consensus in the absence of direct evidence. Their epistemic status differs from the conditional recommendations for Clinical Questions 1 and 2, which rest on systematic evidence assessment. The former are consensus-based and highlight areas where evidence is lacking, while the latter are grounded in formal appraisal of the certainty of evidence.

### PICO 1: In patients with unilateral spatial neglect after stroke, is prism adaptation therapy more effective than other interventions for improving functional outcomes?

The comprehensive and systematic literature search yielded 1,795 records after duplicate removal. During the initial screening phase, four SRs of RCTs met the predefined inclusion criteria. Based on relevance, update of the evidence, and methodological quality, one SR was selected [22], while excluding the remaining reviews. An updated literature search covering the period October 2020 to January 2023 identified ten additional RCTs published after the included SR. Following full-text assessment, four RCTs were deemed eligible [23–26].

The systematic review by Longley et al. (2021) [22] included 65 studies with a total of 1,951 patients with post-stroke spatial neglect. Among the various interventions examined, eight studies (*N* = 257 participants) compared prism adaptation therapy with other treatments, most commonly placebo using neutral lenses.

In the updated literature search, we identified four additional RCTs: two comparing prism lenses with a placebo [24, 27], one with standard occupational therapy [26], and one with neck muscle vibration therapy [23]. These new trials collectively enrolled 472 participants, predominantly males (55%), with a mean age of 63 years (range 51–69 years). The characteristics of these studies, details of the methodological quality assessment, and descriptions of the interventions are reported in the Supplementary Materials.

Outcomes were assessed either at the end of treatment (immediate effects) or at least one month after completion of treatment (persistent effects) and included: (A) Improvement in functional abilities in activities of daily living (ADL), measured using the Catherine Bergego Scale (CBS), the Functional Independence Measure (FIM), and the Barthel Index (BI), where higher scores indicate better performance; (B) Improvement on neuropsychological tests (e.g., line bisection, cancellation tasks, reading tasks). No study evaluated length of hospital stays, health-related quality of life, mood, number of falls, or discharge destination.

**Fig. 1:**
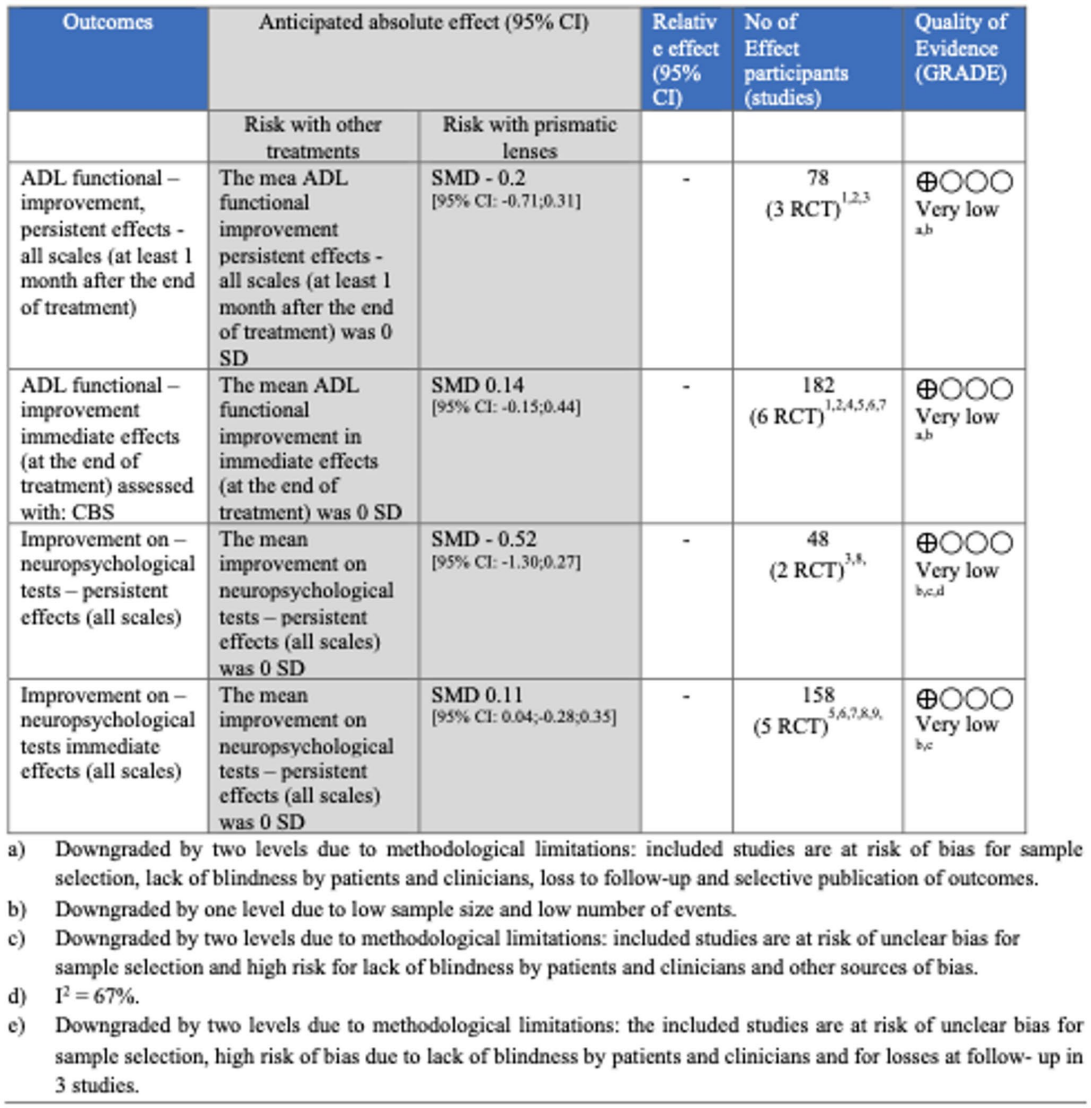
Summary of findings - Question 1 according to the GRADE approach by outcome

For all outcomes, the certainty of the evidence, assessed using the GRADE approach, was rated as very low (see Fig. 1). Only one study [26] reported serious adverse events (four in the intervention group and one in the control group), but none of these events appeared to be related to the use of prism lenses. Complete information on the meta-analysis is provided in the Supplementary Materials.

Three studies could not be included in the meta-analysis, and their results are summarized narratively due to the absence of outcome data. Vilimovsky et al. (2021) [27] conducted a randomized trial in 33 patients with moderate to severe neglect, allocated to intensive prism adaptation therapy or neutral lenses over two weeks (10 sessions in total). Both groups showed functional improvement in ADL, measured with the CBS via the Kessler Foundation Neglect Assessment Process (KF-NAP), and better performance on neuropsychological tests at the different follow-up time points (post-treatment, 2 weeks, and 4 weeks after treatment). However, no between-group differences were observed.

A secondary analysis of an RCT by Mizuno et al. (2021) [24] evaluated the effect of prism adaptation versus neutral lenses on functional outcome (CBS) and anosognosia in 34 patients with neglect recruited from eight rehabilitation centers in Japan. At the end of the intervention, scores on the “gaze orientation” and “personal belongings” items of the CBS were better in the prism group than in the control group; by contrast, no differences were observed for the other CBS items. As regards anosognosia (calculated as the discrepancy between clinician-rated and patient-rated CBS scores), the study indicated that prism adaptation may improve patients’ awareness of their spatial neglect.

Rode et al. (2015) [28] performed an RCT (not included in the Longley et al., 2021 meta-analysis [22]), enrolling 20 patients with moderate to severe neglect, randomly assigned to prism adaptation therapy (*N* = 10) or neutral lenses (*N* = 10). The primary outcome was functional independence in ADL measured with the FIM at 1, 3, and 6 months after treatment. There were no between-group differences at any follow-up. Nonetheless, both groups showed improved functional independence, particularly during the first month.

Based on all this evidence, the Panel developed a conditional recommendation in favor of using specific treatments such as prism adaptation for the rehabilitation of patients with peri-personal neglect (see Table 3). Recommendation (PICO 1 – Conditional recommendation): In patients with peri-personal neglect after stroke, it is indicated to use prism adaptation therapy as part of the rehabilitation program (Certainty of evidence: very low).

**Table 3:** Panel recommendation

Clinical questions	Quality of evidence	Recommendation	Strength of Recommendation
1	Very Low	The Panel indicated rehabilitating patients with peri-personal neglect using specific treatments such as prism adaptation rather than alternative interventions.	Conditional, in favor of the intervention

Findings on patient values and preferences, acceptability and feasibility of the intervention, impact on equity, and resource use were synthesized and incorporated into the EtD framework, available in the Supplementary Materials.

### PICO 2: In patients with unilateral spatial neglect after stroke, should visuospatial training be used compared with other interventions for improving functional outcomes?

After removing duplicates, the systematic search identified 1,795 records. At the end of the selection process, one SR of RCTs fulfilled the inclusion criteria [22]. An updated search (October 2020–January 2023) retrieved one additional RCT, published after the included SR [29].

The systematic review by Longley et al. (2021) [22] included 65 studies involving a total of 1,951 patients with post-stroke spatial neglect. Among the various interventions examined, five studies compared VST programs with alternative treatments. The investigation by Elshout et al. (2021) [29], published after the systematic review, compared a congruent movement training intervention with standard visuospatial training, applied to a group of 20 participants, predominantly males (53%), with a mean age of 58.9 years.

Outcomes assessed across studies, measured either at the end of treatment (immediate effects) or at least one month after treatment completion (persistent effects), included: (A) Improvement in functional abilities in ADL, measured with CBS, FIM, and BI; higher scores indicate better performance; (B) Improvement on neuropsychological tests, such as line bisection, cancellation tasks, and reading tasks. No studies assessed adverse events, length of hospital stay, quality of life, mood, number of falls, or discharge destination.

**Fig. 2:**
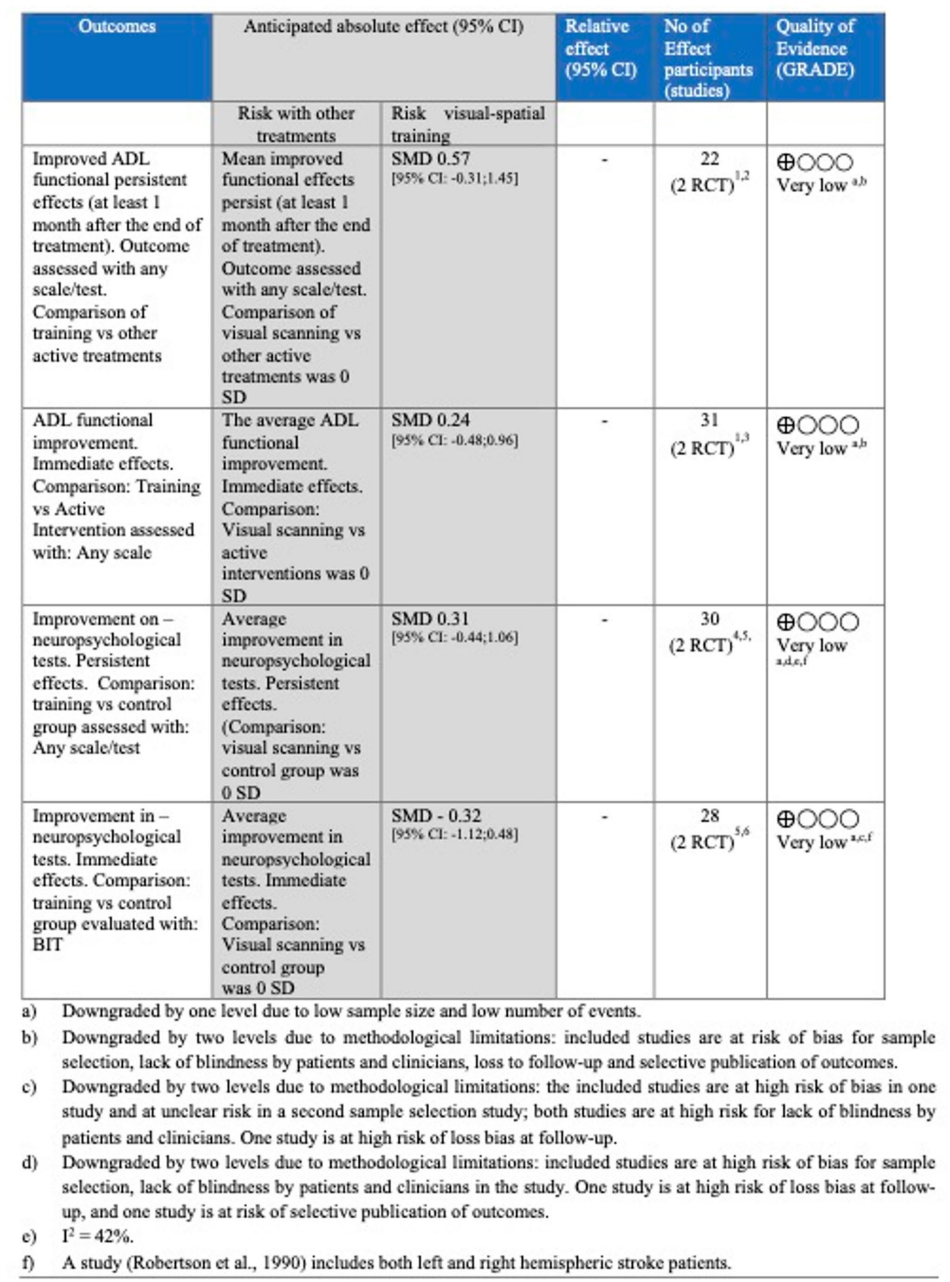
Summary of findings - Question 2 according to the GRADE approach by outcome

The meta-analysis showed overall low effects on ADL and neglect symptoms at the end of treatment (I² = 42%). However, for all outcomes, the certainty of evidence assessed using the GRADE approach was rated as very low (see Fig. 2). Complete information on the meta-analysis is provided in the basic materials.

The study by Elshout et al. (2021) [29] was not included in the meta-analysis due to the absence of outcome data. In the group receiving congruent movement training, post-treatment results showed a positive effect on neglect symptoms across three assessment tests. In contrast, no improvement was observed in the VST group. The authors noted that the study was underpowered to detect statistically significant between-group differences.

Based on all this evidence, the Panel developed a conditional recommendation in favor of using specific treatments such as visuospatial training for the rehabilitation of patients with peri-personal neglect (see Table 4).

**Table 4:** Panel recommendation

Clinical questions	Quality of evidence	Recommendation	Strength of recommendation
2	Very Low	The Panel indicated rehabilitating patients with peri-personal neglect using specific treatments such as visuospatial training rather than alternative interventions.	Conditional, in favor of the intervention

Recommendation (PICO 2 – Conditional recommendation): In patients with peri-personal neglect after stroke, it is indicated to use visuospatial training as part of the rehabilitation program (Certainty of evidence: very low).

Findings related to patient values and preferences, acceptability and feasibility of the intervention, impact on equity, and resource use were narratively summarized and incorporated into the EtD framework, available in the Supplementary Materials.

### PICO 3: In patients with peri-personal neglect after stroke who are candidates for rehabilitation, when should prism adaptation or visuospatial training be initiated to optimize functional outcomes?

The systematic search yielded 1,795 records after duplicate removal. We did not find studies meeting the inclusion criteria during the selection process.

Given the absence of specific literature detailing the optimal timing for initiating neglect rehabilitation, this review referenced broader findings concerning the starting timing of rehabilitation for stroke patients. The literature indicates a lack of consensus and mixed results regarding the efficacy of very early intervention. A narrative review (Coleman et al., 2017) of the AVERT trial indicated that very early and intensive out-of-bed mobilization (within 24 h of stroke onset) resulted in only a modest improvement in walking ability at three months compared to standard care. Furthermore, their review of early rehabilitation for neglect found only one out of three RCTs reporting improved performance (specifically with mirror therapy). Similarly, the Cochrane systematic review by Langhorne et al. (2018), encompassing nine RCTs on very early mobilization (within 48 h), concluded that there were no differences in primary outcomes compared to standard rehabilitation, apart from reduced hospital stays. Conversely, the Australian and New Zealand Living Clinical Guidelines for Stroke Management (2022) issued a strong recommendation against initiating intensive out-of-bed rehabilitation within 24 h of stroke onset. Although Kleim and colleagues (2008) emphasized the relevance of experience-dependent neuroplasticity principles and the potential therapeutic benefit of delivering rehabilitation within optimal temporal windows, evidence for specific early treatments remains inconsistent. For instance, a Cochrane review by Elsner et al. (2020) on non-invasive neuromodulation for neglect found no differences in ADL outcomes based on the timing (acute/subacute versus chronic phase). Collectively, the literature suggests that while the timing of general stroke rehabilitation is critical, the optimal window for *neglect* rehabilitation remains undefined, prompting the need to consider and apply the broader findings cautiously. The panel recommends conducting randomized controlled trials to investigate the impact of the timing of initiation of rehabilitation treatment for spatial neglect.

Indications for good clinical practice, complementary to the formal recommendations above, were formulated for clinical questions 3 and 4 in accordance with the methodological manual of the National Guideline System. They provide operational guidance on specific aspects for which no direct comparative studies among the different therapeutic options were available. These indications, grounded in the extensive clinical experience of the guideline panelists, were unanimously endorsed. For this reason, they are put forward as guidance for good healthcare practice.

### PICO 3: indications for good clinical practice

For patients with peri-personal neglect who are candidates for rehabilitation, the Panel considered it reasonable to start prism adaptation or visuospatial training within 4–7 days after the stroke event, considering individual clinical characteristics within the personalized rehabilitation plan. The Panel also issued a research recommendation encouraging the performance of RCTs aimed at investigating the optimal timing of rehabilitation treatment for spatial neglect.

Good Practice Statement (PICO 3): It is suggested to initiate prism adaptation or visuospatial training within 4–7 days after stroke onset, taking into account the individual clinical characteristics within the personalized rehabilitation plan.

PICO 4: In stroke survivors with peri-personal neglect, are neglect-specific scales (e.g., CBS–KF-NAP, Zoccolotti scale) more accurate than non-specific ADL measures (e.g., Barthel Index, FIM) for evaluating functional status?

This search identified 1,396 studies after removing duplicates, but we did not find studies that met the requirements of the present PICO.

In the framework of assessing the impact of spatial neglect on performance in daily living activities, the expert panel undertook a systematic search for studies comparing neglect-specific instruments, such as the CBS–KF-NAP and the Semi-Structured Neglect Scale developed by Zoccolotti et al. [30, 31], with non-specific functional measures, including the FIM and the BI.

Although the search failed to identify studies meeting the inclusion criteria, the widespread clinical adoption of these tools and the minimal risks associated with their use led the panel to deem it appropriate to formulate two good practice recommendations.

The Catherine Bergego Scale – Kessler Foundation Neglect Assessment Process (CBS–KF-NAP) is a standardized, observational measure used to assess functional aspects of spatial neglect in daily life activities. The CBS evaluates neglect-related behaviors across ten real-life domains. The KF-NAP provides structured scoring guidelines to improve the reliability and ecological validity of the CBS.

Zoccolotti’s scale consists of structured observational tasks designed to identify asymmetric performance in everyday actions, used in clinical research to evaluate personal and extra-personal neglect in stroke patients.

An observational study involving 121 post-stroke patients demonstrated the feasibility of the CBS–KF-NAP in clinical settings, with over 94% of participants completing all scoring categories. The scale showed excellent internal consistency (Cronbach’s α = 0.96) and captured functional aspects of neglect not detected by standard measures such as the FIM or the BI. Overall, the CBS (KF-NAP) has proven sensitive in detecting even mild neglect, showing moderate-to-strong correlations with disability levels on the BI and FIM, particularly the motor items. The scale also resulted to be more sensitive than traditional paper-and-pencil tests, reliably identifying neglect-related difficulties in daily functioning. CBS scores at admission demonstrated predictive value for discharge destination, and the scale allows classification of neglect severity across clinically meaningful categories. A simplified version (sCBS) has also been validated, maintaining strong correlations with the original CBS and the NIHSS while significantly reducing administration time, thus enhancing feasibility in acute stroke care.

The Zoccolotti semi-structured neglect scale has been employed to evaluate personal neglect, in which patients demonstrate asymmetric performance during everyday tasks such as grooming or object exploration. For example, observational research using the Zoccolotti and Judica personal neglect scale revealed distinct recovery trajectories for personal versus extra-personal neglect and highlighted the clinical relevance of neglect in functional outcome studies after stroke [32].

### PICO 4: indications for good clinical practice

For stroke survivors with peri-personal neglect, the Panel recommended using condition-specific instruments to assess activities of daily living (ADL), such as the Catherine Bergego Scale (preferably the KF-NAP version), rather than non-specific measures (e.g., FIM or Barthel Index). In addition, the Panel recommended the use of specific functional scales, such as the Zoccolotti scale, for assessing ADLs. No research recommendation was formulated for PICO 4.

Good Practice Statement (PICO 4): It is suggested to use neglect-specific instruments, particularly the Catherine Bergego Scale (KF-NAP version) and the Zoccolotti semi-structured scale, rather than non-specific ADL measures such as the FIM or the Barthel Index, for assessing functional status in stroke survivors with peri-personal neglect.

## Discussion

Spatial neglect stands as one of the most significant and disabling neuropsychological complications following a stroke, frequently preventing full functional recovery, reducing independence in activities of daily living, and increasing burden on patients, caregivers, and health systems [1, 3]. The present Clinical Practice Guideline represents the first coordinated national initiative for the diagnosis and rehabilitation of post-stroke neglect within the Italian NHS.

The systematic reviews underlying this CPG indicate that the overall certainty of evidence for non-pharmacological interventions in spatial neglect remains very low, despite a relatively large number of trials conducted. The Cochrane review by Longley et al. and its subsequent updates [22, 26] demonstrate that, across multiple intervention approaches, sample sizes are generally small, methodological quality is frequently suboptimal, and outcome measures are heterogeneous, thereby limiting the confidence in estimating treatment effects [27]. Within this context, PAT and VST emerge as two of the most widely studied “specific” rehabilitation techniques for peri-personal neglect; however, their measured benefits remain modest and largely short-term.

For PAT, meta-analyses indicate just modest immediate improvements in ADL and neglect severity compared with control conditions, with no convincing evidence of sustained effects at follow-up. Individual RCTs illustrate both the potential and current limitations. A pilot study in an intensive rehabilitation setting showed comparable functional gains in prism and neutral-lens groups, highlighting the feasibility but not superiority of PAT [27, 28]. Another study [24] reported better performance on selected CBS items and a reduction in anosognosia indices, suggesting that prism procedures may preferentially target awareness and exploratory behavior rather than global independence. Similarly, studies of VST and related visual scanning protocols indicate moderate immediate effects on ADLs and neuropsychological tests. However, the evidence is constrained by small samples, lack of blinding, and absence of robust long-term data [26, 29, 33].

Considering these findings, the conditional recommendations in favor of PAT and VST for peri-personal neglect appear appropriately cautious and aligned with international evidence. Crucially, the CPG does not interpret the very low certainty of evidence as proof of inefficacy, but rather as a reflection of the methodological limitations of existing trials, in line with the conclusions of Cochrane and other reviews. Therefore, within a shared decision-making framework, PAT and VST can be proposed as reasonable, neglect-specific treatment options. Meanwhile, clinicians should transparently communicate that benefits are expected but not yet firmly established.

A critical gap identified by the CPG was the lack of direct evidence regarding the optimal timing for initiating PAT or VST following a stroke. Consequently, the Panel developed a Good Practice Statement (GPS) based on the understanding that the early months post-stroke represent a key temporal window for maximizing therapeutic benefit and capitalizing on experience-dependent neural plasticity principles. The GPS suggests that rehabilitation interventions should reasonably commence within 4–7 days after the stroke event, subject to the patient’s clinical status in the individualized rehabilitation plan.

Similar to the timing question, no pertinent studies directly compared the accuracy of specific versus non-specific ADL scales for measuring neglect outcomes. However, based on high levels of clinical expertise and robust observational evidence, the Panel issued two Good Practice Statements promoting the use of neglect-specific scales.

Specific instruments are required to capture its functional impact on daily life due to the multidimensionality of neglect. The CBS, preferably in the Kessler Foundation Neglect Assessment Process version, and the semi-structured Zoccolotti scale are recommended. Evidence shows that the CBS is highly reliable (Cronbach’s alpha) and more sensitive than traditional paper-and-pencil tests, capturing functional dimensions often missed by generic measures, such as the FIM or the BI. Furthermore, CBS scores predicted relevant outcomes such as discharge destination.

From an organizational standpoint, the widespread presence of neurologists, physiatrists, neuropsychologists, and rehabilitation therapists within stroke care pathways represents an important facilitating factor for the implementation of the recommendations.

The evidence base for the present recommendations is primarily derived from a single systematic review, specifically the Cochrane review by Longley et al. (2021) [22]. While this review demonstrates methodological rigor, it presents several significant limitations that influence the strength and certainty of the recommendations in this CPG. First, the included trials were generally small, with most individual studies enrolling fewer than 50 participants, which limits statistical power and reduces the precision of effect estimates. Second, considerable clinical and methodological heterogeneity was present across studies, including variations in study populations, intervention protocols, outcome measures, and follow-up duration. This heterogeneity complicates the interpretation of pooled estimates and restricts the generalizability of the findings. Third, the risk of bias was frequently assessed as high or unclear in several domains, particularly regarding allocation concealment and blinding of outcome assessors. These methodological issues further diminish confidence in the reported treatment effects.

Taken together, these limitations represent the main drivers of the very low certainty of evidence assigned to all critical outcomes considered in this CPG. The Panel explicitly took these uncertainties into account when formulating the recommendations, choosing conditional rather than strong recommendations. Accordingly, clinicians should interpret the available evidence as supporting PAT and VST as reasonable treatment options, while recognizing that the true magnitude of their benefit remains uncertain.

The authors also acknowledge the possibility that relevant studies may have been published after January 2023. However, in accordance with standard practice for clinical practice guidelines, a formal update of these recommendations is planned within three years of publication.

Finally, the Good Practice Statements formulated for Clinical Questions 3 and 4 should be interpreted with particular caution. Unlike the recommendations for Clinical Questions 1 and 2, these statements are explicitly grounded in expert consensus rather than direct comparative evidence, and therefore carry a different epistemic status. They are intended to provide pragmatic clinical guidance in areas where evidence is lacking, rather than evidence-based recommendations in the GRADE perspective.

## Conclusion

In conclusion, this Guideline represents a first attempt toward standardizing national clinical practice in the diagnosis and treatment of post-stroke neglect, promoting the use of validated assessment instruments, targeted rehabilitation strategies, and a multidisciplinary approach. Implementation of the recommendations, together with outcome monitoring and further research, will help improve the quality of care, the efficiency of the healthcare system, and the quality of life of patients living with neglect.

## Supplementary Information

Below is the link to the electronic supplementary material.

Supplementary Material 1
