# Supplementary material for: From evidence to action: Italian recommendations for the diagnosis and treatment of spatial neglect in stroke patients
Source: Neurol Sci. 2026 May 28;47(6):532. doi: 10.1007/s10072-026-09115-z (PMC13216096; doi:10.1007/s10072-026-09115-z)
Supplement: Supplementary file 1 — Supplementary Material 1 [file 10072_2026_9115_MOESM1_ESM.docx]

**From Evidence to Action: Italian Recommendations for the Diagnosis and Treatment of Spatial Neglect in Stroke Patients**

# **Supplementary Materials**

PICO 1: In patients with unilateral spatial neglect after stroke, is prism adaptation therapy more effective than other interventions for improving functional outcomes?

**Table S1 Summary of the protocol**

| **Population:** | Adults aged ≥18 years with right-hemisphere stroke and peri-personal neglect (i.e., difficulty exploring space whose boundary is defined by upper-limb extension—reaching, grasping), including both egocentric and allocentric forms. |
| --- | --- |
| **Intervention** | Prism lenses. |
| **Comparison** | No treatment; other non-pharmacological treatments. |
| **Outcomes**: | **Efficacy and safety**  **Critical outcomes:** Functional improvement measured using the Catherine Bergego Scale (CBS), Functional Independence Measure (FIM), and Barthel Index; improvement on neuropsychological tests (line bisection, cancellation/barrage, reading); length of hospital stay; quality of life measured with any validated scale.  **Important outcomes:** Mood assessed with rating scales (Beck Depression Scale, Hamilton Depression Scale); number of falls, measured as the number of individuals with fractures; discharge destination (i.e., setting such as home, nursing facility, etc.).  **Other outcomes:** Acceptability (dropout, i.e., early discontinuation for any reason; treatment adherence; satisfaction with treatment); feasibility; patient values; impact on equity of access to care. |
| **Setting** | **Any setting.** |
| **Study design** | **Systematic reviews of RCTs, individual RCT studies. In case of little or no evidence, comparative cohort studies will be considered. No case series and individual cases.** |

**Search strategy**

**Database: Cochrane Library (issue 01, 2023)**

Research Date: 3 gennaio 2023

#1 MeSH descriptor: [Stroke] explode all trees and with qualifier(s): [therapy - TH]

#2 MeSH descriptor: [Stroke Rehabilitation] explode all trees

#3 (neglect or hemineglect):ti,ab,kw

#4 ((perceptual or visuo?spatial or visuo?perceptual or attentional) near/5 (disorder* or deficit* or impairment* or abilit*)):ti,ab,kw

#5 {OR #1-#4}

#6 MeSH descriptor: [Adaptation, Ocular] explode all trees

#7 lenses

#8 (prism or prisms)

#9 (PA or PAT):ti

#10 Visual NEXT Scanning

#11 VST:ti,ab

#12 {OR #6-#11}

#13 #5 AND #12 in Trials

#14 #5 AND #12 in Cochrane Reviews

**Database: Ovid MEDLINE(R) ALL <1946 to January 04, 2023>**

1 Stroke/th [Therapy]

2 Stroke Rehabilitation/

3 exp Perceptual Disorders/

4 (neglect or hemineglect).tw.

5 ((perceptual or visuo?spatial or visuo?perceptual or attentional) adj5 (disorder* or deficit* or impairment* or abilit*)).ti,ab.

6 1 or 2 or 3 or 4 or 5

7 Adaptation, Ocular/

8 Lenses/ or lenses.ti,ab.

9 (prism or prisms).mp.

10 (PA or PAT).ti.

11 visual scanning.mp. or VST.ti.

12 7 or 8 or 9 or 10 or 11

13 6 and 12

14 exp animals/ not humans.sh.

15 13 not 14

**Database: Embase <1974 to 2023 January 03>**

1 cerebrovascular accident/rh, th [Rehabilitation, Therapy]

2 stroke rehabilitation/

3 exp *perception disorder/

4 (neglect or hemineglect).tw.

5 ((perceptual or visuo?spatial or visuo?perceptual or attentional) adj5 (disorder* or deficit* or impairment* or abilit*)).ti,ab.

6 1 or 2 or 3 or 4 or 5

7 visual adaptation/

8 Lenses.mp. or lens/

9 (prism or prisms).mp.

10 (PA or PAT).ti.

11 visual scanning.mp. or VST.ti.

12 7 or 8 or 9 or 10 or 11

13 6 and 12

**Database: APA PsycInfo <1806 to January Week 4 2023>**

1 exp Perceptual Disturbances/

2 exp Perceptual Distortion/

3 exp Sensory Neglect/

4 (neglect or hemineglect).tw.

5 ((perceptual or visuo?spatial or visuo?perceptual or attentional) adj5 (disorder* or deficit* or impairment* or abilit*)).ti,ab.

6 ((stroke* or post stroke or poststroke or post-stroke) and (training or re-training or rehabilitation or intervention or therapy)).tw.

7 1 or 2 or 3 or 4 or 5 or 6

8 Lenses.mp.

9 (prism or prisms).mp.

10 (PA or PAT).ti.

11 visual scanning.mp. or VST.ti,ab.

12 8 or 9 or 10 or 11

13 7 and 12

**Database ati: CINAHL EBSCOHOST**

S11 (S6 AND S10)

S10 (S7 OR S8 OR S9)

S9 TX (visual scanning) OR TI VST

S8 TX (prism or prisms)

S7 (MH "Lenses") OR (TI Lenses OR AB Lenses)

S6 S1 OR S2 OR S3 OR S4 OR S5

S5 (((TI perceptual OR AB perceptual) OR (TI visuo#spatial OR AB visuo#spatial) OR (TI visuo#perceptual OR AB visuo#perceptual) OR (TI attentional OR AB attentional)) N5 ((TI disorder* OR AB disorder*) OR (TI deficit* OR AB deficit*) OR (TI impairment* OR AB impairment*) OR (TI abilit* OR AB abilit*)))

S4 ((TI neglect OR AB neglect) OR (TI hemineglect OR AB hemineglect))

S3 (MH "Stroke Patients")

S2 (MH "Stroke+/TH/RH")

S1 (MH "Perceptual Disorders+")

**Database ati: Web of Science Core Collection**

((TS=(((stroke* or post stroke or poststroke or post-stroke) and (training or re-training or rehabilitation or intervention or therapy)))) OR TS=((neglect or hemineglect))) AND TS=(prism or prisms or “visual scanning”)

**Figure 1 Study selection process**

**Identification of studies through databases**

Records removed prior to screening:

Duplicates removed

(n =1160)

Records identified through databases: Cochrane Library, Medline ed Embase (n = 2955)

**Identification**

Excluded records based on title and abstract = 1782

Records to be evaluated

(n =1796)

table

**Screening**

Records to be evaluated as full text

(n=14)

Full text excluded with reason for exclusion:

(n = 3 RS; 6 RCT)

**Eligibility**

Full text evaluated for eligibility

(n = 14)

**Inclusion**

Inclusion studies = 1 RS; 4 RCT

**Table S2 Excluded studies (Question 1)**

| **References** | **Reason for exclusion** |
| --- | --- |
| Elshout JA, Van der Stigchel S, Nijboer TCW (2021) Congruent movement training as a rehabilitation method to ameliorate symptoms of neglect-proof of concept. Cortex 142:84-93. <https://doi.org/10.1016/j.cortex.2021.03.037> | RCT. Intervention that does not meet the inclusion criteria. |
| Meidian AC, Wahyuddin, Amimoto K. (2022) Rehabilitation interventions of unilateral spatial neglect based on the functional outcome measure: A systematic review and meta-analysis. Neuropsychol Rehabil 32(5):764-793. <https://doi.org/10.1080/09602011.2020.1831554> | SR. Includes the same studies as Longley 2021 |
| Qiu H, Wang J, Yi W, Yin Z, Wang H, Li J. (2021) Effects of prism adaptation on unilateral neglect after stroke: An updated meta-analysis of randomized controlled trials. Am J Phys Med Rehabil. 100(3):259-265. <https://doi.org/10.1097/PHM.0000000000001557> | SR. Includes the same studies as Longley 2021, also non-RCT studies |
| Li J, Li L, Yang Y, Chen S. (2021) Effects of prism adaptation for unilateral spatial neglect after stroke: A systematic seview and meta-analysis. Am J Phys Med Rehabil 100(6):584-591. <https://doi.org/10.1097/PHM.0000000000001598> | SR. Includes the same studies as Longley 2021, moderate methodological quality |
| Bourgeois A, Turri F, Schnider A, Ptak R. (2022) Virtual prism adaptation for spatial neglect: A double-blind study. Neuropsychol Rehabil 32(6):1033-1047. <https://doi.org/10.1080/09602011.2020.1864412> | Study design: is not an RCT |
| Zigiotto L, Damora A, Albini F, Casati C, Scrocco G, Mancuso M, Tesio L, Vallar G, Bolognini N. (2021) Multisensory stimulation for the rehabilitation of unilateral spatial neglect. Neuropsychol Rehabil 31(9):1410-1443. <https://doi.org/10.1080/09602011.2020.1779754> Erratum in: Neuropsychol Rehabil. 2022 Jun;32(5):794-795. | Type of intervention: comparison between prismatic lenses vs visuo-exploratory method |
| Sukumaran S, Sivadasan S, Sakunthala PT, Tandon V, Sarma SP (2020) "Sequential multimodality stimulation" for post-stroke-hemineglect: Feasibility and outcome in a pilot randomized controlled trial. J Clin Neurosci 71:108-112. <https://doi.org/10.1016/j.jocn.2019.08.112> | Type of intervention: Compare a multi-dimensional intervention vs physiotherapy |
| Wyatt LE, Champod AS, Haidar GM, Eskes GA. (2021) Can prism adaptation effects generalize to wheelchair maneuvering? NeuroRehab 49(1):119-128. <https://doi.org/10.3233/NRE-210028> | Population type: Study conducted on a population of healthy people |
| Park JH. (2021) The effects of robot-assisted left-hand training on hemispatial neglect in older patients with chronic stroke: A pilot and randomized controlled trial. Medicine (Baltimore) 100(9):e24781. <https://doi.org/10.1097/MD.0000000000024781> | Type of intervention: Compare a robot-assisted hand intervention training vs visual scanning training using a prism and vibration stimulation applied on the left neck extensors and a middle part of the left forearm |

## **Table S3 Summary of the characteristics of the included studies**

| **Study, design, lens** | **Source of studies** | **Population** | **Experimental intervention/control** | **Inclusion studies** | **Outcomes** |
| --- | --- | --- | --- | --- | --- |
| **Longley 2021**^1^  Study design: SR  Objective: To evaluate the efficacy of non-pharmacological interventions for people with neglect after stroke or other forms of acquired brain injury.  Methodological quality (AMSTAR 2):  High | Cochrane Central Register of Controlled trials  • MEDLINE  • Embase  • PsycINFO until October 2020 | Patients with spatial neglect following stroke N=1951 | I = non-pharmacological interventions.  C = No treatment, other treatment options  Follow up: from discharge to 6 months. | 65 RCT studies:  - Visual intervention:17 studies  **Prismatic lenses* = 8 studies** (257 participants)  Body awareness =12 Studies  Mental function = 7 studies  Movement interventions = 6 studies  Non-invasive brain stimulation (NIBS) =17 study  Electrical stimulation = 8 studies  Acupuncture= 2 studies | Secondary Outcomes:  a. Functional improvement measured with ADL immediately after the end of the intervention,  b. Improved performance in neuropsychological assessment tests,  c. Destination for discharge  d. Equilibrium measured as a persistent effect,  e. Number of falls measured as a persistent effect,  F. Depression and anxiety symptoms measured as a persistent effect,  g. Quality of life and social isolation as a persistent effect,  h. Adverse events (excluding falls). |

* The 8 studies included in Longley et al., 2021 are: Choi et al., 2019; Goedert, 2020; Mancuso et al., 2012; Mizuno et al., 2011; Nys et al., 2008; Rode et al., 2015; Ten Brink et al., 2017; Turton et al. 2010.

**Table S4 Summary of the characteristics of RCTs published after the review by Longley et al. (2021)**

| **Study, Country, study design** | **Inclusion/Exclusion Criteria** | **Comparisons considered** | **Characteristics of the study population** | **Studied outcomes** |
| --- | --- | --- | --- | --- |
| **Choi et al., 2022**  Korea  Multicenter RCT | Diagnosis of stroke based on MRI; K-MMSE≥ 20;  start of stroke at least 6 months earlier; suspected unilateral neglect based on the Motor-Free Visual Perception test. | N=36  Group A: prismatic lenses + neck vibration (n=12)  Group B: neck vibration (n=12)  Group C: prismatic lenses only (n=12)  5 times a week for 50 min/day, for a total of 20 sessions during a 4-week period.  All participants participated in 30 minutes of occupational therapy | times a week for 50 min/day, for a total of 20 sessions during a 4-week period.  All participants participated in 30 minutes of occupational therapy. | At the end of the intervention ADL functional improvement measured with MBI: modified Barthel Index and CBS;  Improvement to line deletion test measured with Albert's test;  Improvement in visuo-perceptual performance measured with the Motor-Free Visual Perception Test. |
| **Longley et al., 2022**  United Kingdom  RCT  **Protocol:**  https://www.isrctn.com/ Ref ISRCTN88395268 | 53 patients with age> 18 years with confirmed ischemic or hemorrhagic stroke; positive screening tests for spatial neglect; had spatial inattention impacting on functional performance; at least one-week post-stroke;  Setting: Inpatient, rehabilitation center. | N = 53  Prismatic lenses (n = 40) vs standard occupational therapy (n = 13)  Daily sessions, 5 days/week, up to 3 weeks. | Average age: 69 (13.3) years,  57% males,  75% ischemic stroke,  91% right hemisphere,  Participants were recruited at 15 (IQR: 11, 21) post-stroke days (median value). | *Assessment at baseline, at 3 and 12 weeks after the start of the intervention:*  Improvement at the tests: Hearts cancellation test: a subtest of the  Oxford Cognitive Screen; Star cancellation: a subtest of the Behavioural  Inattention Test; Reading test; Kessler Foundation Neglect Assessment  Process (KF-NAP).  *Only at 12 weeks:*  - Nottingham Extended Activities of Daily  Living scale (NEADL);  - Patient Reported Evaluation of Cognitive State (PRECiS): measure of the perception of the impact of cognitive problems,  - EQ5D5L2,  - Length of stay and place of destination,  - Modified Rankin score (mRS),  - Adverse events.  *Outcomes measured on carer at 12 weeks:*  1. Carer experience scale  2. Modified carer strain index  3. Self-reported informal carer health service use |
| **Vilimovsky et al., 2021**  Czech Republic  RCT  Protocol: NR | (a) age between 18 and 75 years, (b)  With acquired brain injuries (head trauma or stroke); c) brain injuries for no more than one month after hospital discharge; (d) able to participate in intensive rehabilitation therapy; (e) presence of severe or moderate spatial neglect as indicated by the Catherine Bergego Scale test (CBS *>* 10) via the Kessler Foundation Neglect Assessment Process (KF-NAP1) | N=33  PAT (n=12)vs Sham (n=11)  10-sessions for 2 weeks  Session duration:20' | **PAT**  Type of brain injury (stroke/other forms):11/1  **Neglected side** (left/right):11/1  Average age: 51.5 years (47.5–55)  **Sham**  Median age: 58 years (53–61)  Brain injury type (stroke/other forms):10/1  **Neglected side** (left/right):9/2  **Time post injury/stroke at admission**  **PAT=** 58 (38.5–74) days  Control= 48 (35–79) days  **Time post injury/stroke at the first PAT session** (in days):  PAT=76 (69–133.5) days  Control= 70 (62–97) days | Improvement in neglect symptoms assessed with CBS via Kessler Foundation Neglect Assessment Process  (KF-NAP1) (final score from 0 to 30; a positive score indicates the presence of neglect); Bell Test; Line bisection; Scene copying test.  The assessment was made at baseline (T1), post-treatment (T2), 2 weeks post-treatment (T3), and 4 weeks post-treatment (T4). |
| **Mizuno et al., 2021**  Japan  (RCT, secondary analysis by Mizuno 2011) | 38 participants (444 enrolled) recruited from rehabilitation departments of 8 hospitals. Patients must have had a first right hemisphere stroke event no more than 3 months ago; BIT-verified neglect condition | N = 38  PAT vs Neutral lenses  20 sessions (bi-daily, 5 days for 2 weeks) | Prismatic lenses = 15  Average age: 64 ± 11.5 years  Time since event: 19.6 ± 5.78 days  Control group=19  Average age: 66.5 ± 7.7 years  Time from stroke event: 27.1 ± 14.2 days | Functional improvement measured with CBS.  Self-awareness(anosognosia) score was calculated by recording the difference between the observer’s assessment scores and the patient’s self-assessment scores. The anosognosia score  is of a positive value if the patient has a higher CBS score than the rater.  The assessment was made at baseline (T0), at two weeks after treatment (T1), and at discharge (T2 = follow-up). |
| **Chen et al., 2021**  United States  Retrospective study | 1568 patients with neglect (CBS>0) of which 666 patients received at least 1 PAT session and 902 untreated. | N = 312  PAT (n = 156) vs No PAT (n = 156)  Group PAT:  8-12 Sessions: 231 patients (34.7%),  8 Sessions: 34 patients  (14.7%),  9 Sessions: 28 patients (12.1%),  10 Sessions: 162 patients (70.1%),  11 Sessions:7 patients (3.0%),  12 Sessions: 1 patient (0.4%). | PAT Group  Mean age = 69 (61.5-77) years  Women = 82 (52.6%)  Diagnosis = 139 (89.1%) stroke  Group no PAT  Mean age = 70 (61-77.5) years  Women = 70 (44.9%)  Diagnosis = 136 (87.2%) stroke. | Functional improvement measured with FIM,  Hospital discharge rate. |

Legenda: PAT=Prism adaptation training; CBS = Catherine Bergego Scale; MRI = Magnetic Resonance; K-MMSE= Mini-Mental Status Examination, Korean version; MBI: modified Barthel Index; FIM = Functional Independence Measure

**References (studies included)**

- Choi HS, Lee BM (2022) A complex intervention integrating prism adaptation and neck vibration for unilateral neglect in patients of chronic stroke: A randomised controlled trial. Int J Environ Res Public Health 19(20):13479. <https://doi.org/10.3390/ijerph192013479>

- Chen P, Diaz-Segarra N, Hreha K, Kaplan E, Barrett AM (2021) Prism adaptation treatment improves inpatient rehabilitation outcome in individuals with spatial neglect: A retrospective matched control study. Arch Rehabil Res Clin Transl. 3(3):100130. <https://doi.org/10.1016/j.arrct.2021.100130>

- Longley V, Hazelton C, Heal C, Pollock A, Woodward-Nutt K, Mitchell C, Pobric G, Vail A, Bowen A (2021) Non-pharmacological interventions for spatial neglect or inattention following stroke and other non-progressive brain injury. Cochrane Database Syst Rev 7(7):CD003586. <https://doi.org/10.1002/14651858.CD003586.pub4>

- Mizuno K, Tsujimoto K, Tsuji T. 2021 Effect of prism adaptation therapy on the activities of daily living and awareness for spatial neglect: A secondary analysis of the randomized, controlled trial. Brain Sci. 11(3):347. <https://doi.org/10.3390/brainsci11030347>

- Rode G, Lacour S, Jacquin-Courtois S, Pisella L, Michel C, Revol P, et al. (2014) Long-term sensorimotor and therapeutical effects of a mild regime of prism adaptation in spatial neglect. A double-blind RCT essay. Ann. Phys Rehab Med 58:40–53.
- Vilimovsky T, Chen P, Hoidekrova K, Petioky J, Harsa P. (2021) Prism adaptation treatment to address spatial neglect in an intensive rehabilitation program: A randomized pilot and feasibility trial. PLoS One. 16(1):e0245425. <https://doi.org/10.1371/journal.pone.0245425>

## **Table S5 Methodological quality assessment of SRs (AMSTAR 2 checklist)**

| **Study** | **Item 1** | **Item 2** | **Item 3** | **Item 4** | **Item 5** | **Item**  **6** | **Item 7** | **Item**  **8** | **Item**  **9** | **Item 10** | **Item 11** | **Item 12** | **Item 13** | **Item 14** | **Item 15** | **Item 16** | **Global judgement** |
| --- | --- | --- | --- | --- | --- | --- | --- | --- | --- | --- | --- | --- | --- | --- | --- | --- | --- |
| Longley et al., 2021 | Yes | Yes | Yes | Yes | Yes | Yes | Yes | Yes | Yes | Yes | Yes | Yes | Yes | Yes | Yes | Yes | High |
| Li 2021 | Yes | No | No | Yes partially | Yes | Yes | No | Yes | Yes | No | Yes | Yes | No | No | Yes | Yes | Low |

**AMSTAR checklist (**Shea BJ, Reeves BC, Wells G et al. (2020) AMSTAR 2: Tool for the critical evaluation of systematic reviews of randomized trials and/or non-randomized studies on the effectiveness of health interventions. Evidence 12(2): E1000206; Italian version by the GIMBE Foundation).

1. Do the research questions and review inclusion criteria include elements of the PICO?

2. Does the systematic review explicitly state that the methods were defined before it was conducted, justifying all significant violations of the Protocol?

3. Do the authors justify the choice of the design of the studies included in the review?

4. Have the authors carried out a systematic search of the literature?

5. Was the selection of studies carried out by at least two authors independently?

6. Was the data extraction carried out by at least two authors independently?

7. Do the authors provide the list of excluded studies justifying the reasons?

8. Do the authors describe the included studies in sufficient detail?

9. Did the authors use an adequate method to analyze the risk of bias of the individual studies included in the review?

10. Do the authors report the sources of funding for the studies included in the review?

11. If a meta-analysis was conducted, did the authors use appropriate methods for statistical combination of results?

12. If a meta-analysis has been conducted, do the authors analyze the potential impact of the risk of bias of individual studies in the results of the meta-analysis or in the other summaries of evidence?

13. Do the authors take into account the risk of bias in individual studies when interpreting/discussing the results of the review?

14. Do the authors satisfactorily explain and discuss any heterogeneity observed in the results of the review?

15. If a meta-analysis was performed, did the authors adequately explore publication bias and discuss the potential impact on review outcomes?

16. Did the authors report any potential sources of conflict of interest, including any funding received to conduct the review?

**Figure 2 Assessment of risk of bias of RCTs, in accordance with the Cochrane Risk of Bias tool (Longley et al., 2021 and subsequent updates)**


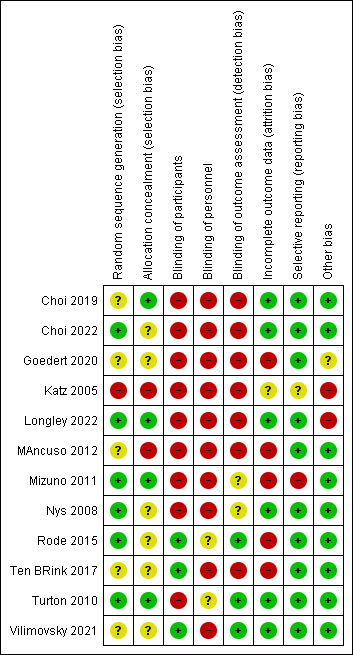


**Table S6 Interventions and comparisons considered in the included studies**

| **Study** | **Experimental intervention (1)** | **Experimental intervention (2)** | **Control intervention** |
| --- | --- | --- | --- |
| Choi et al., 2019 | Prismatic lenses | Prismatic lenses + Electrical stimulation | Electrical stimulation |
| Choi et al., 2022* | Prismatic lenses |  | Neck stimulation |
| Goedert et al., 2020 | Prismatic lenses |  | Standard treatment |
| Longley et al., 2022* | Prismatic lenses |  | Standard occupational therapy |
| Mancuso et al., 2012 | Prismatic lenses |  | sham |
| Mizuno et al., 2011 | Prismatic lenses |  | sham |
| Mizuno et al., 2021* | Prismatic lenses |  | sham |
| Nys et al., 2008 | Prismatic lenses |  | sham |
| Rode et al. 2015 | Prismatic lenses |  | sham |
| Turton et al., 2010 | Prismatic lenses |  | sham |
| Ten Brink et al., 2017 | Prismatic lenses |  | sham |
| Vilimovsky et al., 2021* | Prismatic lenses |  | sham |

* New studies; Mizuno et al. (2021) reports results on the same sample as Mizuno 2011.

**Figure 3 - Improved ADL functional persistent effects (at least 1 month after the end of treatment)**


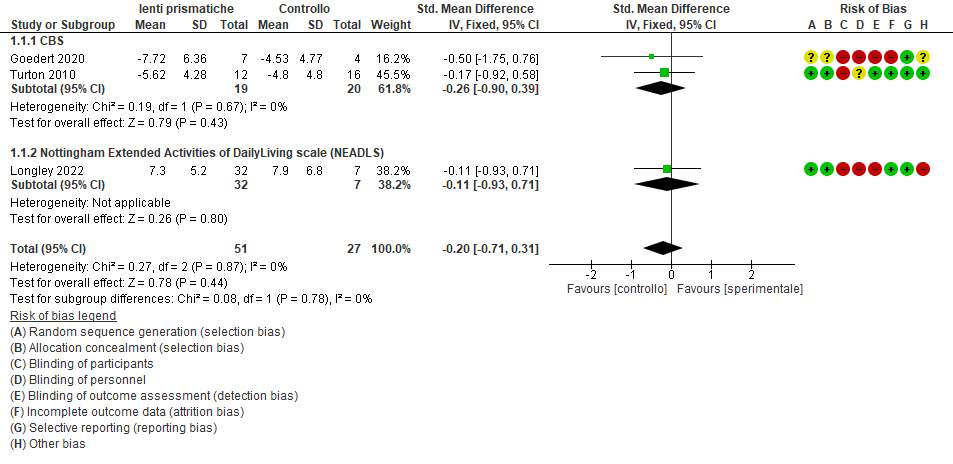


**Figure 4 - Functional improvement ADL immediate effects (at the end of treatment)**


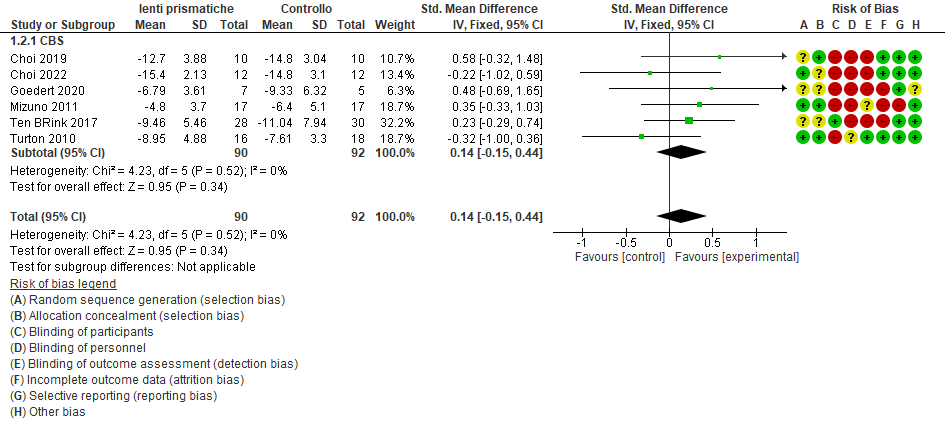


**Figure 5 - Improvement on neuropsychological tests - persistent effects**


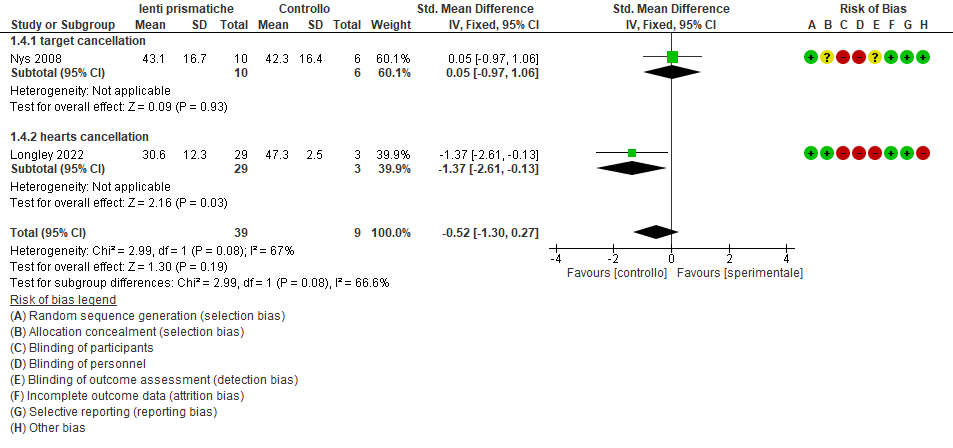


**Figure 6 - Improvement on neuropsychological tests immediate effects (all scales).
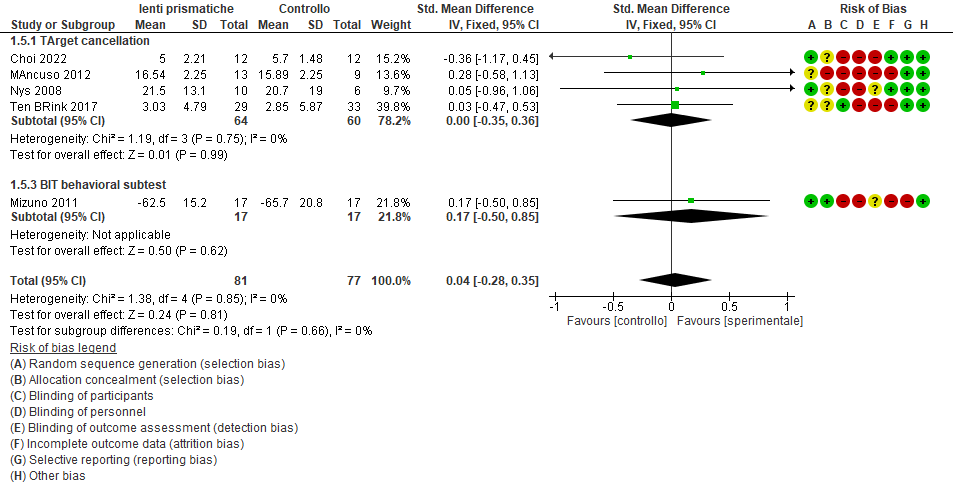
**

## **Evidence to Decision Framework**

|  | |
| --- | --- |
| **PICO 1: In patients with unilateral spatial neglect after stroke, is prism adaptation therapy more effective than other interventions for improving functional outcomes?** | |
| **Population:** | Adults (>18 years) with post-stroke spatial hemineglect |
| **Intervention:** | Prismatic lenses |
| **Comparison:** | No treatment, other treatments |
| **Outcomes:** | Improved ADL functional persistent effects (at least 1 month after the end of treatment); Functional improvement ADL immediate effects (at the end of treatment); Improvement on neuropsychological tests - persistent effects; Improvement on neuropsychological tests immediate effects (all scales). |
| **Setting:** | Any setting |
| **Outlook:** | National Health Service (NHS) |
| **Conflicts of interest:** | No relevant or potentially relevant interest has been identified. All panel members present at the meeting voted, determining the direction and strength of the recommendation. |

**Rating**

| Problem  Is the problem a priority? | | |
| --- | --- | --- |
| Ratings | Searching for evidence | Additional considerations |
| ○ No  ○ Probably not  ○ Probably yes  **● Yes**  **○** Varied  ○ Don't know | Neglect (or Unilateral Spatial Hemineglect) is a neuropsychological syndrome characterized by the inability of the patient to perceive or pay attention to objects, people, representations, located in a visual hemifield (usually contralateral to the lesion), and to act on that side of the space (Kerkhoff et al., 2001; Husain, 2008). Neglect is usually associated with a lesion of the right hemisphere, especially in the lower part of the parietal lobe, and affects the left half of the personal or extra-personal space. Neglect simultaneously encompasses several factors: perception (hemi-inattention), action (motor negligence), and representation (cognitive functions). However, there can be several parts of space that can be affected by Neglect: personal space (space used by one's body); peri-personal space (space accessible by hand); extrapersonal space (space verifiable only by sight or hearing) (Spaccavento et al., 2017).  This disorder, whose symptoms are complex, brings with it a series of consequences that affect the performance of daily activities (dressing, personal hygiene), difficulty in reading, disorientation during walking, as well as reduced adherence to the proposed treatments, preventing the patient's general functional recovery. The presence of neglect is also associated with a longer hospital stay, less likelihood of returning to one's home after hospital discharge, and a greater risk of falls (Chen et al. 2015; Campbell et al., 2010; Bosma et al., 2020).  There is not much data available on the occurrence of neglect. A recent systematic review (Esposito et al.,2021) estimated the prevalence of neglect after unilateral stroke from 20-40%, regardless of the type of lesion, the methodology followed for diagnosis, and the event of stroke.  Rehabilitation of neglect is essential to effectively restore the functions affected in this type of syndrome. In addition to this, the complexity of the symptoms of neglect makes it necessary to design specific rehabilitation interventions for the different existing types of this syndrome, in order to ensure the possibility that the patient achieves a certain degree of functional independence in daily activities (Li et al., 2015; Spaccavento et al., 2017).  Despite the wide spectrum of rehabilitation treatments currently used, it is not yet possible to formally recommend a rehabilitation technique. Among the proposed rehabilitation approaches are prismatic adaptation and visuo-exploratory training, although the available evidence is still uncertain, especially with regard to long-term clinical efficacy.  Several international guidelines (Winstein et al., 2016, NICE, 2019; VA/DoD, 2019) suggest the use of different rehabilitation treatments but do not provide guidance on which interventions should be considered first-line, second-line, and third-line.    **References**   1. Bosma MS, Nijboer TWC, Caljouw MAA, Achterberg WP (2020) Impact of visuospatial neglect post-stroke on daily activities, participation and informal caregiver burden: A systematic review. Ann Phys Rehab Med 63(4):344-358. 2. Campbell GB, Matthews JT (2010). An integrative review of factors associated with falls during post-stroke rehabilitation. J Nurs Scholarsh 42(4):395–404. <https://doi.org/10.1111/j.1547-5069.2010.01369.x> 3. Chen P, Hreha K, Kong Y, Barrett AM (2015) Impact of spatial neglect in stroke rehabilitation: evidence from the setting of an inpatient rehabilitation facility. Arch Phys Med Rehabil 96(8):1458–1466. <https://doi.org/10.1016/j.apmr.2015.03.019> 4. -Esposito E, Shekhtman G, Chen P. (2021) Prevalence of spatial neglect post-stroke: A systematic review. Ann Phys Rehabil Med. 64(5):101459. <https://doi.org/10.1016/j.rehab.2020.10.010> 5. Kerkhoff G (2001) Spatial hemineglect in humans. Prog Neurobiol 63(1):1-27. <https://doi.org/10.1016/s0301-0082(00)00028-9> 6. Husain M. (2008) Hemispatial neglect. Handb Clin Neurol 88:359-372. <https://doi.org/10.1016/S0072-9752(07)88018-3> 7. Li, K., Malhotra P.A. Spatial neglect. Pract Neurol;15 (2015) 333–339. <https://doi.org/10.1136/practneurol-2015-001115> 8. Mazzucchi, A. (2016). La riabilitazione neuropsicologica. Premesse teoriche e applicazioni cliniche. Editore: Edra 9. Spaccavento S, Cellamare F, Falcone R, Loverre A, Nardulli R (2017) Effect of subtypes of neglect on functional outcome in stroke patients. Ann Phys Rehabil Med 60(6):376–381. <https://doi.org/10.1016/j.rehab.2017.07.245> 10. -The Management of Stroke Rehabilitation Work Group. VA/DoD CLINICAL PRACTICE GUIDELINE FOR THE MANAGEMENT OF STROKE REHABILITATION. Available at: <https://www.healthquality.va.gov/guidelines/Rehab/stroke/VADoDStrokeRehabCPGFinal8292019.pdf> 11. National Clinical Guideline Centre (NICE). Stroke Rehabilitation in Adults. London: NICE, 2013. Aggiornamento 2019. Clinical guideline no. 162. Available at: <https://www.nice.org.uk/guidance/cg162> (Last access: 6 February 2023). 12. -Winstein CJ, Stein J, Arena R, Bates B, Cherney LR, Cramer SC, et al. (2016) Guidelines for adult stroke rehabilitation and recovery: a guideline for healthcare professionals from the American Heart Association/American Stroke Association. Stroke 47(6): e98–e169. |  |
| **Desirable effects**  How considerable are the expected desirable effects? | | |
| Ratings | Searching for evidence | Additional considerations |
| ○ Irrelevant  **● Small**  ○ Moderate  ○ Large  ○ Vary  ○ Don't know | **Comparison:** prismatic lenses vs other treatments  **Source:** Longley et al. (2021) and subsequent updates   \| Outcomes \| Anticipated absolute effect^*^ (95% CI) \| \| Relative Effect (95% CI) \| No of participants(studies) \| Quality of Evidence (GRADE) \| \| --- \| --- \| --- \| --- \| --- \| --- \| \| Risk with other treatments \| Risk with prismatic lenses \| \| ADL functional improvement, persistent effects-all scales (at least 1 month after the end of treatment) \| The mean ADL functional improvement persistent effects-all scales (at least 1 month after the end of treatment) was **0** SD \| SMD **0.2 SD less** (0.71 less than 0.31 greater) \| - \| 78 (3 RCT)^1,2,3^ \| ⨁◯◯◯ Very low ^a,b^ \| \| ADL functional improvement immediate effects (at the end of treatment) assessed with: CBS \| The mean ADL functional improvement in immediate effects (at the end of treatment) was **0** SD \| SMD **0.14 SD greater** (0.15 less than 0.44 greater) \| - \| 182 (6 RCT)^1,2,4,5,6,7^ \| ⨁◯◯◯ Very low ^a,b^ \| \| Improvement on neuropsychological tests - persistent effects (all scales) \| The mean improvement on neuropsychological tests - persistent effects (all scales) was **0** \| SMD **0.52 less** (1.3 less than 0.27 greater) \| - \| 48 (2 RCT)^3,8^ \| ⨁◯◯◯ Very low ^b,c,d^ \| \| Improvement on neuropsychological tests immediate effects (all scales) \| The mean improvement on neuropsychological tests of immediate effects (all scales) was **0** SD \| SMD **0.11 SD greater** (0.23 less than 0.45 greater) \| - \| 134 (5 RCT)^5,6,7,8,9^ \| ⨁◯◯◯ Very low ^b,e^ \|  1. Lowered by two levels due to methodological limitations: included studies are at risk of bias for sample selection, lack of blindness by patients and clinicians, loss to follow-up and selective publication of outcomes, 2. Lowered by one level due to low sample size and low number of events, 3. Lowered by two levels due to methodological limitations: included studies are at risk of unclear bias for sample selection and high risk for lack of blindness by patients and clinicians and other sources of bias, 4. I^2^ = 67% 5. Lowered by two levels due to methodological limitations: the included studies are at risk of unclear bias for sample selection, high risk of bias due to lack of blindness by patients and clinicians and for losses at follow-up in 3 studies.   **References**   1. Goedert KM, Chen P, Foundas AL, Barrett AM (2020) Frontal lesions predict response to prism adaptation treatment in spatial neglect: a randomised controlled study. Neuropsychol Rehab 30(1):32–53. <https://doi.org/10.1080/09602011.2018.1448287> 2. Turton AJ, O'Leary K, Gabb J, Woodward R, Gilchrist I. (2010) A single blinded randomised controlled pilot trial of prism adaptation for improving self-care in stroke patients with neglect. Neuropsychol Rehab *20*(2):180–196. <https://doi.org/10.1080/09602010903040683> 3. Longley V, Woodward-Nutt K, Turton AJ, Stocking K, Checketts M, Bamford A, Douglass E, Taylor J, Woodley J, Moule P, Vail A, Bowen A. (2022) A study of prisms and therapy in attention loss after stroke (SPATIAL): A feasibility randomised controlled trial. Clin Rehabil 37(3), 381-393. <https://doi.org/10.1177/02692155221134060> 4. Choi HS, Kim DJ, Yang YA. (2019) The effect of a complex intervention program for unilateral neglect in patients with acute-phase stroke: a randomized controlled trial. Osong Pub Health Res Perspect 10(5):265-273. <https://doi.org/10.24171/j.phrp.2019.10.5.02> 5. Mizuno K, Tsuji T, Takebayashi T, Fujiwara T, Hase K, Liu M. (2011) Prism adaptation therapy enhances rehabilitation of stroke patients with unilateral spatial neglect: a randomized, controlled trial. Neurorehab Neur Rep 25(8):711-720. <https://doi.org/10.1177/1545968311407516> 6. Ten Brink AF, Visser-Meily JMA, Schut MJ, Kouwenhoven M, Eijsackers ALH, Nijboer TCW (2017) Prism adaptation in rehabilitation? No additional eCects of prism adaptation on neglect recovery in the subacute phase poststroke: a randomized controlled trial. Neurorehab Neur Rep 31(12):1017-1028. <https://doi.org/10.1177/1545968317744277> 7. Choi H-S, Lee B-M (2022) A complex intervention integrating prism adaptation and neck vibration for unilateral neglect in patients of chronic stroke: A randomised controlled trial. Int J Environ Res Public Health, 19(20):13479. <https://doi.org/10.3390/ijerph192013479> 8. Nys GMS, De Haan EHF, Kunneman A, De Kort PLM, Dijkerman HC. (2008) Acute neglect rehabilitation using repetitive prism adaptation: a randomized placebo-controlled trial. Restor Neurol Neurosci 26(1):1-12. [https://doi.org/10.3233/RNN-2008-0041](https://doi.org/10.3233/RNN-2008-00417) 9. Mancuso M, Pacini M, Gemignani P, Bartalini B, Agostini B, Ferroni L, et al. (2012) Clinical application of prismatic lenses in the rehabilitation of neglect patients. A randomized controlled trial. Eur J Phys Rehabil Med, 48(2), 197-208.   **Other data**  Two studies were not considered in the meta-analysis. The results are described narratively.  **Vilimovsky et al. (2021)** reported the results of a randomized trial conducted on 33 patients with moderate to severe neglect, assigned to intensive rehabilitation treatment with prismatic lenses or the use of neutral glasses for a period of two weeks, for a total of 10 sessions. For both groups, a functional improvement in daily activities measured with the CBS via Kessler Foundation Neglect Assessment Process (KF-NAP) and an improvement in performance on the different neuropsychological tests at the various follow-ups (after treatment, at 2 weeks and at 4 weeks after treatment) were reported. However, no difference was observed between the groups.  A secondary analysis of an RCT study (Mizuno et al., 2021) investigated the effect of rehabilitation treatment with prismatic lenses compared to the use of neutral glasses on functional improvement (measured with CBS) and self-awareness (anosognosia) in patients with neglect. The study was conducted on 34 patients enrolled from eight rehabilitation centers in Japan.  The results show that, at the end of the intervention, the scores obtained in the "gaze orientation" and "personal belongings" dimensions of the CBS scale were better in the group treated with prismatic lenses than in the control group. For the other dimensions of CBS, no differences were observed between the two groups. Regarding anosognosia, calculated by recording the difference between the scores obtained through the assessment by the clinician and that of the patient, the study suggests that treatment with prismatic lenses may improve awareness about the problem of spatial neglect.  **Rode et al. (2015)**, an RCT study (not included in the meta-analysis by Longley et al., 2021) conducted in a French rehabilitation center, involved 20 patients with moderate to severe neglect, assigned to rehabilitation treatment with prismatic lenses (n=10) or use of neutral glasses (n=10). The primary outcome was functional improvement in performing daily activities as measured by FIM at 1-, 3-, and 6-month post-treatment. The study shows no differences between the two groups at the various follow-ups, but both groups improve their functional independence, especially in the first month.  **References**   1. Rode G, Lacour S, Jacquin-Courtois S, Pisella L, Michel C, Revol P, ... Rossetti, Y (2015) Long-term sensorimotor and therapeutical effects of a mild regime of prism adaptation in spatial neglect. A double-blind RCT essay. Ann Phys Rehab Med 58(2):40-53. 2. Vilimovsky T, Chen P, Hoidekrova K, Petioky J, Harsa P (2021) Prism adaptation treatment to address spatial neglect in an intensive rehabilitation program: A randomized pilot and feasibility trial. PLoS One 16(1):e0245425. <https://doi.org/10.1371/journal.pone.0245425> | The literature search identified a retrospective observational study (Chen et al., 2021) that assessed functional improvement, measured with the Functional Independence Measure (FIM) scale, and discharge rate in a sample of 312 stroke patients recruited from 14 rehabilitation centers and treated with prismatic or untreated lenses. The study observed that patients who received PAT showed total functional improvement, especially in the cognitive area, compared to patients who did not receive PAT. On the other hand, no difference was observed between the two groups with regard to the motor area and the rate of discharge from the rehabilitation center (OR = 1.33; P = 0.258). |
| **Undesirable effects**  How considerable are the expected undesirable effects? | | |
| Ratings | Searching for evidence | Additional considerations |
| **● Irrelevant**  **○ Small**  **○ Moderate**  **○ Large**  **○ Vary**  **○ Don't know** | The identified studies reported no data on adverse events due to treatment with prismatic lenses. |  |
| **Certainty of evidence**  What is the overall certainty of the evidence of efficacy and safety? | | |
| Ratings | Searching for evidence | Additional considerations |
| **● Very low**  **○ Low**  **○ Moderate**  **○ High**  **○ No study included** | The quality of the evidence was found to be very low for all the outcomes evaluated.  Quality was lowered mainly due to the risk of bias in RCTs (selection bias) and inaccuracy due to a very low sample size. |  |
| **Values**  Is there uncertainty or variability about how important people may consider the main outcomes? | | |
| Ratings | Searching for evidence | Additional considerations |
| ○ Important uncertainty  or variability  ○ Probably important uncertainty or variability  **● Probably not important uncertainty or variability**  ○ No major uncertainty or variability | No studies have been identified that have reported data on the value that people with neglect place on rehabilitation treatment outcomes. | In the absence of available studies, the panel believes that the outcomes considered in the research are shared by patients and their families. |
| Balance of effects  Does the balance between desirable and undesirable effects favor intervention or comparison? | | |
| Ratings | Searching for evidence | Additional considerations |
| ○ Promotes comparison  ○ Likely favors comparison  ○ Favors neither comparison nor treatment  ○ Probably favors treatment  **● Favors treatment**  ○ Varies  ○ Don't know | In favor of treatment. |  |
| Resources Needed  How large are the resources needed (costs)? | | |
| Ratings | Searching for evidence | Additional considerations |
| ○ Very high costs **● Moderate costs**  ○ Irrelevant costs and savings  ○ Moderate savings  ○ High savings○ Varies  ○ Don't know | Economic Evaluation Report. |  |
| Quality of evidence in relation to the resources needed  What is the quality of the evidence in relation to the resources needed (costs)? | | |
| Ratings | Searching for evidence | Additional considerations |
| ○ Very low  ○ Low  ○ Moderate  ○ High  ● **No study included** | Economic Evaluation Report |  |
| Cost Effectiveness  Does the cost-effectiveness analysis favor intervention or comparison? | | |
| Ratings | Searching for evidence | Additional considerations |
| ○ Promotes comparison ○ Likely favors comparison  ○ Favors neither comparison nor treatment  ○ Probably favors treatment  ○ Favors treatment ○ Varies  ● **Don't know** | Economic Evaluation Report. |  |
| **Fairness**  What would be the impact in terms of equity? | | |
| Ratings | Searching for evidence | Additional considerations |
| ○ Reduces equity  ○ Likely reduces equity  ○ Probably no impact  **● Likely improves equity**  **○** Improves equity  ○ Varies  ○ I don't know | No studies have been identified that have reported data on the impact of rehabilitation treatment with prismatic lenses on equity. | See the data on the survey on access to services available in Italy |
| **Acceptability**  Is the intervention acceptable for the main stakeholders? | | |
| Ratings | Searching for evidence | Additional considerations |
| ○ No  ○ Probably not **● Probably yes**  **○** Yes  ○ Varies  ○ I don't know | The search of the literature led to the identification of only one study, described narratively below.  **Longley et al. (2022)**, a randomized controlled trial conducted in the United Kingdom, evaluated the efficacy and acceptability of a prismatic lens-based rehabilitation intervention (n = 40) compared to standard occupational therapy (n = 13). The participants, mostly male, with ischemic stroke and positive screening tests for spatial neglect, were enrolled and assigned to the two treatments after a median time from the stroke event of 15 days (median value).  Among the results of the study, the authors report that the intervention with prismatic lenses was overall considered acceptable by the participants. Through questionnaires and interviews with both patients and caregivers, the rehabilitation treatment was found to be stimulating and challenging in the various sessions. The results of the interviews with therapists also confirmed this finding, pointing out that some patients had reported increased awareness following treatment.  Seven of the 31 patients who completed the questionnaire reported finding training sessions with prismatic lenses tiring, highlighting the need for a quieter environment for greater concentration.  **Reference**  Longley V, Woodward-Nutt K, Turton AJ, Stocking K, Checketts M, Bamford A, Douglass E, Taylor J, Woodley J, Moule P, Vail A, Bowen A. (2022) A study of prisms and therapy in attention loss after stroke (SPATIAL): A feasibility randomised controlled trial. Clin Rehabil 37(3):381-393. <https://doi.org/10.1177/02692155221134060> |  |
| Feasibility  Is the implementation of the intervention feasible? | | |
| Ratings | Searching for evidence | Additional considerations |
| ○ No  ○ Probably not  ○ Probably yes  **● Yes**  ○ Varies  ○ I don't know | The literature search led to the identification of seven studies, described narratively below.  **Gillen et al. (2022)**, a retrospective cohort study, evaluated the feasibility of a rehabilitation treatment with prismatic lenses (PAT) and the capacity of the intervention to improve symptoms of spatial neglect and functional abilities in patients with right hemispheric stroke and spatial neglect.  From the medical records of 524 hospitalized patients, from 2016 to 2019, 39 patients who had completed 4 or more treatment sessions with prismatic lenses were selected. In the same period, and based on the score at CBS and FIM, the control group was selected, treated with other rehabilitation therapies (visual scanning, limb activation).  Regarding the improvement of neglect symptoms, the study showed a positive association between the CBS score at admission and an improvement in CBS among patients who received the PAT (Spearman's ρ = 0.680, P < 0.001). Stratified based on the score at CBS at discharge, the patients were classified into patients with high, moderate, and low severity of neglect. Patients included in the category with greater severity reported a benefit from prismatic lens therapy; the median CBS score at discharge for patients with severe neglect was 15, IQR: 13–17 vs. 23, IQR: 21–26 (p < 0.05). There were no between-group differences in discharge scores in patients with low and moderate severity neglect.  Many patients received the recommended 10 sessions of PAT (mean number of completed sessions=8.6)  Patients assigned to the PAT had a greater number of days of hospitalization than patients who did not receive PAT. The median length of stay for patients with severe neglect was, in fact, 25 days vs 16 days for patients in the control group (P < 0.05). Patients with moderate neglect treated with PAT also reported a higher number of days of hospitalization (28 vs. 19 days, P < 0.05).  **Hreha et al. (2018)** conducted a prospective observational study to evaluate the feasibility and efficacy of PAT to improve spatial and motor functions in multiple stroke survivors. Thirteen participants, from an inpatient rehabilitation facility (IRF), were assigned to 10 daily sessions with PAT, while another 13 patients received only standard care, making up the comparison group. The study reported that participants completed the entire treatment protocol, no adverse events were reported, nor difficulties in carrying out the instructions received.  The same author reported the data of this study one year after the intervention (Hreha et al., 2020). Of the 26 participants, three (60%) assigned to PAT and two (50%) to the control group still had spatial neglect.  **Hreha et al. (2020) and Hreha et al. (2022)** reported the results of a study on the feasibility and sustainability of two care protocols for spatial neglect, the Kessler Foundation Neglect Assessment Process (KF-NAP) for the assessment of neglect using the Catherine Bergego Scale (CBS) in the course of daily activities, and the Kessler Foundation Prism Adaptation Treatment (KF-PAT), a standardized protocol for treatment with prismatic lenses.  The study included a training period on the two protocols for the physiotherapists involved in the research, followed by a 6-month implementation phase of the protocols themselves. During this period, among the patients evaluated, 26 had a diagnosis of neglect, and 10 were treated with PAT.  Overall, fidelity to both protocols was maintained; no participants demonstrated difficulty in following treatment instructions, nor reported fatigue or discomfort.  The study also investigated possible barriers and factors facilitating the application of care protocols involving 16 U.S. rehabilitation centers and 169 occupational therapists. The main barriers in administering PAT, identified through interviews, were: (1) short length of stay; (2) other priority clinical objectives; and (3) lack of appropriately trained health personnel to administer all planned sessions of PAT.  **Chen et al. (2017)** reported the results of an online survey aimed at healthcare professionals to learn about their knowledge, opinions and obstacles on rehabilitation treatments for patients with neglect. In particular, a clinical case was presented to the professionals, and they were asked to plan a treatment plan in different post-stroke phases, assuming an ideal scenario and one adapted to clinical practice. One hundred and twenty-seven professionals from various medical disciplines in 23 countries responded to the survey. Based on years of experience, the professionals were divided into two subgroups: a subgroup consisting of 30 highly specialized experts with at least 10 years of clinical or teaching experience and at least five scientific publications on neglect; a second subgroup of 97 experts with less experience. Among the treatments chosen by the experts, visual scanning, "Active limb activation", prismatic adaptation and "sustained attention training" were the first five selected in the conditions of an ideal scenario. In both scenarios, more treatments were chosen in the acute (2 weeks to 3 months) and subacute (3 to 12 months post-stroke) phase, compared to the early (<2 weeks post-stroke) or chronic (>12 months post-trauma) phases.  As for the obstacles perceived by the professionals involved in the survey, 16.3% of professionals stated that they had no obstacles, and 15.8% did not consider spatial neglect a priority. About 44.6% of participants reported that they did not have the necessary time to treat patients with neglect and 37.6% that they did not have the necessary instrumentation. Other answers concerned the difficulty of interdisciplinary relationships, the absence of standardized protocols, and the lack of effectiveness of treatments.  **Evald et al. (2020)** reported the results of an online survey conducted in Denmark aimed at healthcare professionals on the diagnosis and treatment of neglect. Among 525 professionals involved in the survey, 411 (78.3%) reported that, in their clinical practice, treatments for neglect were usually delivered. A deeper analysis of the responses showed that a small proportion of practitioners (n = 78, 14.9%) reported that no neglect treatments were offered in their clinical setting, and a smaller number (n = 36, 6.9%) were unaware of the availability of these treatments.  With regard to the professional figures involved, occupational therapists were among the most involved, followed by physiotherapists, especially in public health services. About a third of respondents reported the figure of nurses and social workers, more frequent in the hospital setting. Less involved were psychologists, speech therapists and doctors.  With regard to treatment initiation, participants employed in a hospital setting reported timely initiation of treatment for neglect (within one month of the event) compared to that reported by professionals employed in the private sector (3 to 6 months post-event).  The frequency of treatment was also higher in the hospital and private setting (once a day) than in the municipal setting (2-3 times a week). The duration of the sessions reported was 21-30 minutes for the hospital and private settings and 31-40 minutes for the municipal setting.  The majority of participants reported that personal experience and contact with colleagues were the basis for their choice of treatment. Few practitioners have reported using national guidelines or other studies as a source of evidence when choosing the type of treatment.  **References**   1. Chen P, Pitteri M, Gillen G, Ayyala H. (2018) Ask the experts how to treat individuals with spatial neglect: a survey study. Disabil Rehabil. 40(22):2677-2691. <https://doi.org/10.1080/09638288.2017.1347720> 2. -Chen P, Hreha K, Gonzalez-Snyder C, et al. (2022) Impacts of prism adaptation treatment on spatial neglect and rehabilitation outcome: Dosage matters. Neurorehab Neural Repair 36(8):500-513. https://doi.org/[10.1177/15459683221107891](https://doi-org.bibliosan.idm.oclc.org/10.1177/15459683221107891) 3. Evald L, Wilms IL, Nordfang M. (2020) Treatment of spatial neglect in clinical practice: A nationwide survey. Acta Neurol Scand. Jan;141(1):81-89. <https://doi.org/10.1111/ane.13179> 4. Gillen RW, Harmon EY, Weil B, Fusco-Gessick B, Novak PP, Barrett AM (2022) Prism adaptation treatment of spatial neglect: Feasibility during inpatient rehabilitation and identification of patients most likely to benefit. Front Neurol. 13:803312. <https://doi.org/10.3389/fneur.2022.803312> 5. Hreha K, Gillen G, Noce N, Nilsen D. (2018) The feasibility and effectiveness of using prism adaptation to treat motor and spatial dysfunction in stroke survivors with multiple incidents of stroke. Top Stroke Rehabil. 25(4):305-311. <https://doi.org/10.1080/10749357.2018.1437937> 6. Hreha K, Rich T, Wong JA (2020) One-year follow-up study on community dwelling multiple stroke survivors with spatial neglect. Occup Ther Health Care 34(1):48-61. <https://doi.org/10.1080/07380577.2020.1737894> 7. -Hreha K, Chen P, LaRosa J, Santos C, Gocon C; Barrett AM. (2020) Implementing a rehabilitation protocol for spatial neglect assessment and treatment in an acute care hospital. J Acute Care Phys Ther 11(2)59-69. <https://doi.org/10.1097/JAT.0000000000000117> 8. Hreha K, Barrett AM, Gillen RW, Gonzalez-Snyder C, Masmela J, Chen P. (2022) The implementation process of two evidence-based protocols: A spatial neglect network initiative. Front. Health Serv 2: 839517. <https://doi.org/10.3389/frhs.2022.839517> |  |

| **Summary of judgments** | **Ratings** | | | | | | |
| --- | --- | --- | --- | --- | --- | --- | --- |
| **Priority of the Problem** | No | Probably not | Probably yes | **Yes** |  | Varies | Don't know |
| **Desirable effects** | Irrelevant | Small | **Moderate** | Large |  | Varies | Don't know |
| **Undesirable effects** | Large | Moderate | Small | **Irrelevant** |  | Varies | Don't know |
| **Quality of Evidence** | **Very low** | Low | Moderate | High |  |  | No studies included |
| **Values** | Important uncertainty or variability | Probably important uncertainty or variability | **Probably not important uncertainty or variability** | No major uncertainty or variability |  |  |  |
| **Balance of effects** | In favor of confrontation | Probably in favor of comparison | It does not favor either discussion or intervention | Probably in favor of the intervention | **In favor of intervention** | Varies | Don't know |
| **Resources Needed** | Large costs | **Moderate costs** | Negligible costs and savings | Moderate savings | Large savings | Varies | Don't know |
| **Quality of evidence in relation to the resources needed** | Very low | Low | Moderate | High |  |  | **No studies included** |
| **Cost Effectiveness** | In favor of confrontation | Probably in favor of comparison | Not favor either comparison or treatment | Probably in favor of the intervention | In favor of intervention | Varies | **No studies included** |
| **Fairness** | Reduced | Probably reduced | Probably no impact | **Probably increased** | Increased | Varies | Don't know |
| **Acceptability** | No | Probably not | **Probably yes** | Yes |  | Varies | Don't know |
| **Feasibility** | No | Probably not | Probably yes | **Yes** |  | Varies | Don't know |

**Type of recommendation**

| Strong recommendation against intervention | Conditional recommendation against intervention | Conditional recommendation in favor of both intervention and discussion | Conditional recommendation in favor of intervention | Strong recommendation in favor of intervention |
| --- | --- | --- | --- | --- |
| ○ | ○ | ○ |  | ○ |

**Conclusion**

| Recommendations |
| --- |
| Recommendation (PICO 1 – Conditional recommendation): In patients with peri-personal neglect after stroke, it is indicated to use prism adaptation therapy as part of the rehabilitation program (Certainty of evidence: very low). |
|  |
| Justification |
|  |

| Considerations for subgroups |
| --- |
|  |
| Implementation considerations |
| The training of health personnel is an important factor in the effectiveness of the intervention; therefore, it is suggested to provide training for dedicated staff |

| Monitoring and evaluation |
| --- |
|  |
| Research priorities |
| It would be desirable to conduct multicenter, comparative studies in patients in both the acute and chronic phases, with follow-ups at various post-treatment intervals (greater than six months). Studies should evaluate clinical outcomes such as the number of falls, length of stay, and quality of life.  It would also be desirable, in conducting comparative studies, to better define the types and severity of neglect.  The panel suggests conducting qualitative studies on neglected patients and caregivers to explore the acceptability of rehabilitation treatments. |

##

## **Table S6 Table of evidence (GRADE)**

**Comparison:** Prismatic lenses versus other treatments for patients with spatial hemineglect (neglect)

**Setting:** Any

**Reference:** Longley et al. (2021) and subsequent updates.

| **Certainty assessment** | | | | | | | **No of patients** | | **Effect** | | **Quality of evidence** | **Importance** |
| --- | --- | --- | --- | --- | --- | --- | --- | --- | --- | --- | --- | --- |
| **No of studies** | **Study design** | **Risk of distortion** | **Lack of reproducibility of results** | **Lack of generalizability** | **Inaccuracy** | **Additional considerations** | **Prismatic lenses** | **Other treatments** | **Relative (95% IC)** | **Absolute (95% CI)** |  |  |
| **Functional improvement ADL persistent effects-all scales (at least 1 month after end of treatment)** | | | | | | | | | | | | |
| 3 ^1,2,3^ | Randomized trials | very serious ^a^ | not important | not important | Serious ^b^ | none | 51 | 27 | - | SMD **0.2 SD less**  (0.71 less than 0.31 greater) | ⨁◯◯◯ Very low | critical |
| **Functional improvement ADL immediate effects (at the end of treatment) (assessed with: CBS)** | | | | | | | | | | | | |
| 6 ^1,2,4,5,6,7^ | Randomized trials | very serious ^a^ | not important | not important | Serious ^b^ | none | 90 | 92 | - | SMD **0.14 SD greater**  (0.15 less than 0.44 greater) | ⨁◯◯◯ Very low | critical |
| **Improvement on neuropsychological tests - persistent effects (all scales)** | | | | | | | | | | | | |
| 2 ^3,8^ | Randomized trials | very serious ^c^ | Serious ^d^ | not important | Serious ^b^ | none | 39 | 9 | - | SMD **0.52 less**  (1.3 less than 0.27 greater) | ⨁◯◯◯ Very low | critical |
| **Improvement on neuropsychological tests – immediate effects (all scales)** | | | | | | | | | | | | |
| 5 ^5,6,7,8,9^ | Randomized trials | very serious ^e^ | not important | not important | Serious ^b^ | none | 69 | 65 | - | SMD **0.11 SD greater**  (0.23 less than 0.45 greater) | ⨁◯◯◯ Very low | critical |

**CI:** Confidence interval; **SMD:** Standardised mean difference

**Explanations**

a. Lowered by two levels due to methodological limitations: included studies are at risk of bias for sample selection, lack of blindness by patients and clinicians, loss to follow-up and selective publication of outcomes.

b. Lowered by one level due to low sample size and low number of events.

c. Lowered by two levels due to methodological limitations: included studies are at risk of unclear bias for sample selection and high risk for lack of blindness by patients and clinicians, and other sources of bias.

d. I2 = 67%.

e. Lowered by two levels due to methodological limitations: the included studies are at risk of unclear bias for sample selection, high risk of bias due to lack of blindness by patients and clinicians and for losses at follow-up in 3 studies.

**References**

1. Goedert KM, Chen P, Foundas AL, Barrett AM (2020) Frontal lesions predict response to prism adaptation treatment in spatial neglect: a randomised controlled study. Neuropsychol Rehab 30(1):32–53. <https://doi.org/10.1080/09602011.2018.1448287>
2. Turton AJ, O'Leary K, Gabb J, Woodward R, Gilchrist I. (2010) A single blinded randomised controlled pilot trial of prism adaptation for improving self-care in stroke patients with neglect. Neuropsychol Rehab *20*(2):180–196. <https://doi.org/10.1080/09602010903040683>
3. Longley V, Woodward-Nutt K, Turton AJ, Stocking K, Checketts M, Bamford A, Douglass E, Taylor J, Woodley J, Moule P, Vail A, Bowen A. (2022) A study of prisms and therapy in attention loss after stroke (SPATIAL): A feasibility randomised controlled trial. Clin Rehabil 37(3), 381-393. <https://doi.org/10.1177/02692155221134060>
4. Choi HS, Kim DJ, Yang YA. (2019) The effect of a complex intervention program for unilateral neglect in patients with acute-phase stroke: a randomized controlled trial. Osong Pub Health Res Perspect 10(5):265-273. <https://doi.org/10.24171/j.phrp.2019.10.5.02>
5. Mizuno K, Tsuji T, Takebayashi T, Fujiwara T, Hase K, Liu M. (2011) Prism adaptation therapy enhances rehabilitation of stroke patients with unilateral spatial neglect: a randomized, controlled trial. Neurorehab Neur Rep 25(8):711-720. <https://doi.org/10.1177/1545968311407516>
6. Ten Brink AF, Visser-Meily JMA, Schut MJ, Kouwenhoven M, Eijsackers ALH, Nijboer TCW (2017) Prism adaptation in rehabilitation? No additional eCects of prism adaptation on neglect recovery in the subacute phase poststroke: a randomized controlled trial. Neurorehab Neur Rep 31(12):1017-1028. <https://doi.org/10.1177/1545968317744277>
7. Choi H-S, Lee B-M (2022) A complex intervention integrating prism adaptation and neck vibration for unilateral neglect in patients of chronic stroke: A randomised controlled trial. Int J Environ Res Public Health, 19(20):13479. <https://doi.org/10.3390/ijerph192013479>
8. Nys GMS, De Haan EHF, Kunneman A, De Kort PLM, Dijkerman HC. (2008) Acute neglect rehabilitation using repetitive prism adaptation: a randomized placebo-controlled trial. Restor Neurol Neurosci 26(1):1-12. [https://doi.org/10.3233/RNN-2008-0041](https://doi.org/10.3233/RNN-2008-00417)
9. Mancuso M, Pacini M, Gemignani P, Bartalini B, Agostini B, Ferroni L, et al. (2012) Clinical application of prismatic lenses in the rehabilitation of neglect patients. A randomized controlled trial. Eur J Phys Rehabil Med, 48(2), 197-208.

PICO 2: In patients with unilateral spatial neglect after stroke, should visuospatial training be used compared with other interventions for improving functional outcomes?

**Table S7 Summary of the protocol**

| **Population:** | Adults over 18 years of age affected by right hemispheric stroke and peripersonal neglect (i.e., with difficulty in exploring the space whose boundary is defined by the extension of the upper limb-reaching, grasping), with both egocentric and allocentric forms. |
| --- | --- |
| **Intervention** | Training visuo-spatial |
| **Comparison** | No treatment, other non-pharmacological treatments |
| **Outcomes**: | Critical outcomes: Functional improvement measured with CBS, FMI and BI, Improvement on neuropsychological tests (line bisection test, cancellation/barrage, reading), Length of stay, Quality of life measured with any validated scale.  Important: Mood measured with rating scales (Beck Depression scale, HAD scale), Number of falls to the ground measured as the number of people with fractures, Fate at discharge.  *Other outcomes:* Acceptability (dropout, adherence to treatment, satisfaction with treatment), Feasibility, Values, Fairness. |
| **Setting** | Any setting |
| **Study design** | Systematic reviews of RCTs, individual RCTs. In case of little or no evidence, comparative cohort studies will be considered. No case series and individual cases. |

##

**Search strategy**

Research Date: 3 January 2023

**Cochrane Library (issue 01, 2023)**

#1 MeSH descriptor: [Stroke] explode all trees and with qualifier(s): [therapy - TH]

#2 MeSH descriptor: [Stroke Rehabilitation] explode all trees

#3 (neglect or hemineglect):ti,ab,kw

#4 ((perceptual or visuo?spatial or visuo?perceptual or attentional) near/5 (disorder* or deficit* or impairment* or abilit*)):ti,ab,kw

#5 {OR #1-#4}

#6 MeSH descriptor: [Adaptation, Ocular] explode all trees

#7 lenses

#8 (prism or prisms)

#9 (PA or PAT):ti

#10 Visual NEXT Scanning

#11 VST:ti,ab

#12 {OR #6-#11}

#13 #5 AND #12 in Trials

#14 #5 AND #12 in Cochrane Reviews

**Database: Ovid MEDLINE(R) ALL <1946 to January 04, 2023>**

1 Stroke/th [Therapy]

2 Stroke Rehabilitation/

3 exp Perceptual Disorders/

4 (neglect or hemineglect).tw.

5 ((perceptual or visuo?spatial or visuo?perceptual or attentional) adj5 (disorder* or deficit* or impairment* or abilit*)).ti,ab.

6 1 or 2 or 3 or 4 or 5

7 Adaptation, Ocular/

8 Lenses/ or lenses.ti,ab.

9 (prism or prisms).mp.

10 (PA or PAT).ti.

11 visual scanning.mp. or VST.ti.

12 7 or 8 or 9 or 10 or 11

13 6 and 12

14 exp animals/ not humans.sh.

15 13 not 14

**Database: Embase <1974 to 2023 January 03>**

1 cerebrovascular accident/rh, th [Rehabilitation, Therapy]

2 stroke rehabilitation/

3 exp *perception disorder/

4 (neglect or hemineglect).tw.

5 ((perceptual or visuo?spatial or visuo?perceptual or attentional) adj5 (disorder* or deficit* or impairment* or abilit*)).ti,ab.

6 1 or 2 or 3 or 4 or 5

7 visual adaptation/

8 Lenses.mp. or lens/

9 (prism or prisms).mp.

10 (PA or PAT).ti.

11 visual scanning.mp. or VST.ti.

12 7 or 8 or 9 or 10 or 11

13 6 and 12

**Database: APA PsycInfo <1806 to January Week 4 2023>**

1 exp Perceptual Disturbances/

2 exp Perceptual Distortion/

3 exp Sensory Neglect/

4 (neglect or hemineglect).tw.

5 ((perceptual or visuo?spatial or visuo?perceptual or attentional) adj5 (disorder* or deficit* or impairment* or abilit*)).ti,ab.

6 ((stroke* or post stroke or poststroke or post-stroke) and (training or re-training or rehabilitation or intervention or therapy)).tw.

7 1 or 2 or 3 or 4 or 5 or 6

8 Lenses.mp.

9 (prism or prisms).mp.

10 (PA or PAT).ti.

11 visual scanning.mp. or VST.ti,ab.

12 8 or 9 or 10 or 11

13 7 and 12

**CINAHL EBSCOHOST**

S8 (S6 AND S7)

S7 TX (visual scanning) OR TI VST

S6 S1 OR S2 OR S3 OR S4 OR S5

S5 (((TI perceptual OR AB perceptual) OR (TI visuo#spatial OR AB visuo#spatial) OR (TI visuo#perceptual OR AB visuo#perceptual) OR (TI attentional OR AB attentional)) N5 ((TI disorder* OR AB disorder*) OR (TI deficit* OR AB deficit*) OR (TI impairment* OR AB impairment*) OR (TI abilit* OR AB abilit*)))

S4 ((TI neglect OR AB neglect) OR (TI hemineglect OR AB hemineglect))

S3 (MH "Stroke Patients")

S2 (MH "Stroke+/TH/RH")

S1 (MH "Perceptual Disorders+")

**Web of Science Core Collection for:** ((TS=(((stroke* or post stroke or poststroke or post-stroke) and (training or re-training or rehabilitation or intervention or therapy)))) OR TS=((neglect or hemineglect))) AND TS=(“visual scanning”)

**Figure 7 Study selection process**

**Identification of studies through databases**

Records removed prior to screening:

Duplicates removed

(n =1160)

Records identified through databases: Cochrane Library, Medline ed Embase (n = 2955)

**Identification**

Excluded records based on title and abstract = 1789

Records to be evaluated

(n =1795)

**Screening**

Records to be evaluated as full text

(n = 5)

Full text excluded with reason for exclusion: (n = 3 RCT)

Full text evaluated for eligibility (n = 5)

**Eligibility**

Included studies =1 RS; 1 RCT

**Inclusione**

**Table S8. Excluded studies**

| **Reference** | **Reason for exclusion** |
| --- | --- |
| Batool S, Zafar H, Gilani SA, Ahmad A, Hanif A (2022) Effects of visual scanning exercises in addition to task specific approach on balance and activities of daily livings in post stroke patients with eye movement disorders: a randomized controlled trial. BMC Neurol 22(1):312. <https://doi.org/10.1186/s12883-022-02843-7> | Type of intervention that does not meet the inclusion criteria |
| Zigiotto L, Damora A, Albini F, Casati C, Scrocco G, Mancuso M, Tesio L, Vallar G, Bolognini N. (2021) Multisensory stimulation for the rehabilitation of unilateral spatial neglect. Neuropsychol Rehabil 31(9):1410-1443. <https://doi.org/10.1080/09602011.2020.1779754>  Erratum in: Neuropsychol Rehabil. 2022 32(5):794-795. | Type of intervention: comparison between prismatic lenses vs visuo-exploratory method |
| Park JH. (2021) The effects of robot-assisted left-hand training on hemispatial neglect in older patients with chronic stroke: A pilot and randomized controlled trial. Medicine (Baltimore) 5;100(9):e24781.<https://doi.org/10.1097/MD.0000000000024781> | Intervention Type: Compare a robot-assisted hand intervention training vs Visual-spatial training |

## **Table S9 Summary of the characteristics of the included studies**

| **Study, design, lens** | **Source of studies** | **Population** | **Experimental intervention/control** | **Inclusion studies** | **Outcomes** |
| --- | --- | --- | --- | --- | --- |
| **Longley 2021**^1^  Study design: RS  Objective: To evaluate the efficacy of non-pharmacological interventions for people with neglect after stroke or other forms of acquired brain injury | Cochrane Central Register of Controlled trials  • MEDLINE  • Embase  • PsycINFO until October 2020 | Patients with spatial neglect following stroke  N=1951 | I = non-pharmacological interventions.  C=no treatment, other treatment options  Follow up: from discharge to 6 months | 65 RCT studies, of which:  -**Visual intervention:17*** studies  Prismatic lenses = 8 studies;  Body awareness=12 studies  Mental function= 7 study  Movement interventions= 6 studi  Non-invasive brain stimulation (NIBS)=17 study  Electrical stimulation= 8 studies  acupuncture= 2 studies | Primary Outcome:  Functional improvement, measured with ADL, persistent for at least 1 month after the end of the intervention  Secondary outcomes:  at. Functional improvement measured with ADL immediately after the end of the intervention.  b. Improved performance on neuropsychological assessment tests  c. destination for discharge  d. Equilibrium measured as a persistent effect  e. Number of falls measured as a persistent effect  f. depression and anxiety symptoms measured as a persistent effect  g. Quality of life and social isolation as a persistent effect  h. Adverse events (excluding falls) |

* The studies included in the review are: Cherney 2002, Cottam et al. 1987, Ferreira et al. 2011, Katz et al. 2005, Lukkainen-Makkula et al. 2009, Robertson et al. 1990; van Wyk et al., 2014.

**Table S10 Summary of the characteristics of RCTs published after the review by Longley et al., 2021**

| **Study,**  **Country, study design** | **Inclusion/Exclusion Criteria** | **Comparisons considered** | **Characteristics of the study population** | **Studied outcomes** |
| --- | --- | --- | --- | --- |
| **Elshout et al., 2021**  Netherlands  RCT  **Protocollo:** https://www.trialregister.nl/trial/6818) | Pcs enrolled from rehabilitation centers with visuo-spatial neglect after stroke as indicated by CBS ≥ 6 test, omission difference of 2 or more between contralesional and ipsilesional side at  shape cancellation test, ≥2 of 4 lines deviant on line bi-section test (8 repetitions of 4 different lines).  Pcs with TBI, severe aphasia, and/or insufficient level of understanding were excluded. | Congruent Movement Training vs visual spatial training  Each patient received 10 sessions, lasting 30 minutes of VST or CMT (5 hours in total). | Total subjects= 20  Age:  VST=58.7 (4.2) years  CMT=59.2 (4) years  Gender (males):  VST=40%  CMT=66.7%  Time since event (days):  VST=76.8 (9)  CMT= 102.6 (21.8) | Overall improvement in neglect symptoms calculated from the combination of 1) difference in the number of omissions (between  contralesional and ipsilesional side of the figure) at the cancellation test; 2) the mm deviation from center on the line bi-section test, plus 3) score at the CBS test. |

Legenda: CMT= Congruent movement training; VST=visual scanning training; CBS=Catherine Bergego Scale.

**References**

- Elshout JA, Van der Stigchel S, Nijboer TCW (2021) Congruent movement training as a rehabilitation method to ameliorate symptoms of neglect-proof of concept. Cortex 142:84-93. <https://doi.org/10.1016/j.cortex.2021.03.037>

- Longley V, Hazelton C, Heal C, Pollock A, Woodward-Nutt K, Mitchell C, Pobric G, Vail A, Bowen A (2021) Non-pharmacological interventions for spatial neglect or inattention following stroke and other non-progressive brain injury. Cochrane Database Syst Rev 7(7):CD003586. <https://doi.org/10.1002/14651858.CD003586.pub4>

**Figure 8 Assessment of risk of bias of RCTs, in accordance with the Cochrane Risk of Bias tool (Longley et al., 2021, and subsequent updates)**


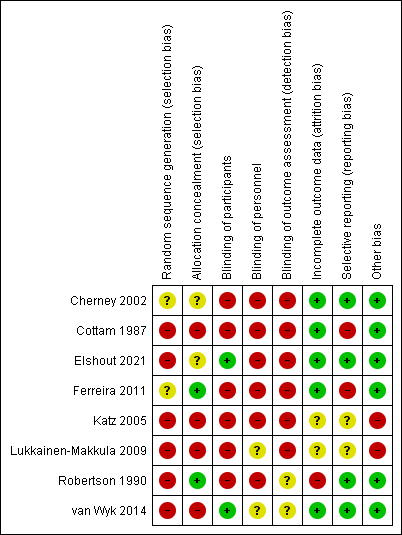


**Table S11 Interventions and comparisons considered in the included studies**

| **Study** | **Experimental intervention (1)** | **Experimental intervention (2)** | **Control intervention** |
| --- | --- | --- | --- |
| Cherney et al. 2003 | visual scanning |  | Attention control |
| Cottam et al. 1987 | visual scanning |  | No intervention |
| Elshout et al., 2021 | congruent movement training |  | Visual scanning |
| Ferreira et al. 2011 | Visual scanning | Mental function |  |
| Katz et al. 2005 | visual scanning | Mental function (VR training) |  |
| Luukkainen-Makkula et al. 2009 | visual scanning | Body awareness (limb activation) |  |
| Robertson et al. 1990 | visual scanning |  | Attention control |
| van Wyk et al. 2014 | visual scanning |  | Attention control |

**Figure 9 - Improved ADL functional persistent effects (at least 1 month after the end of treatment)**


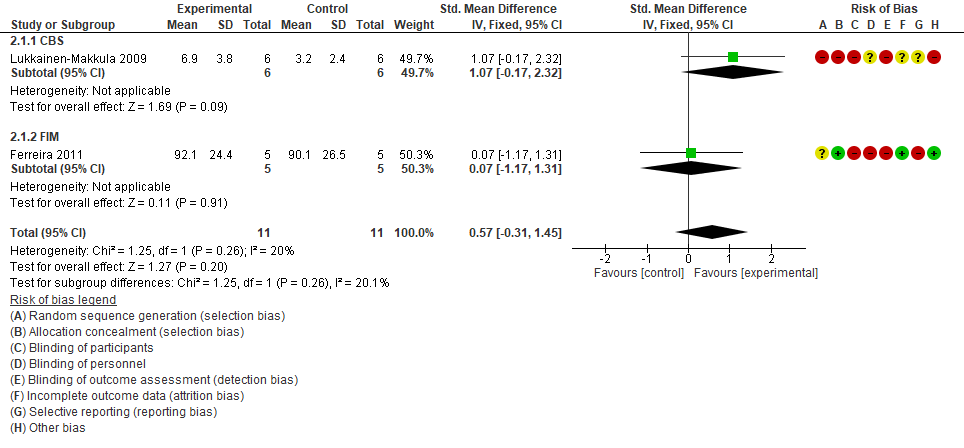


**Figure 10 - Improvement on neuropsychological tests immediate effects (all scales).**


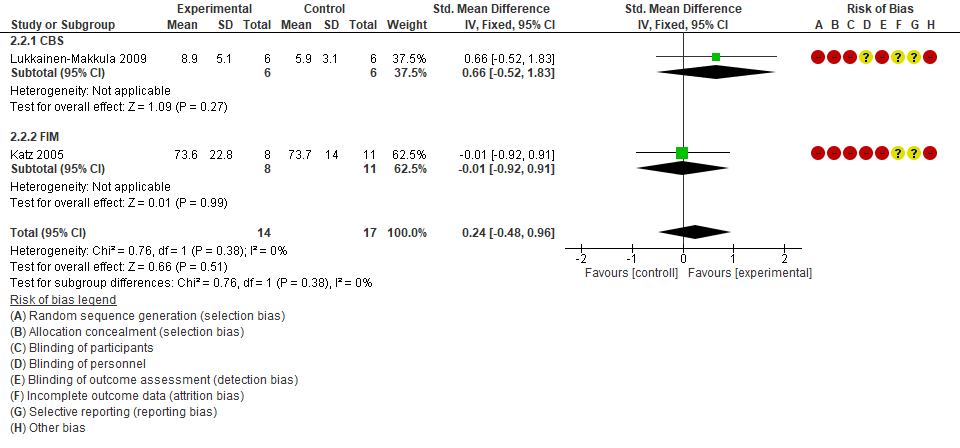


**Figure 11 - Improvement on neuropsychological tests - persistent effects**


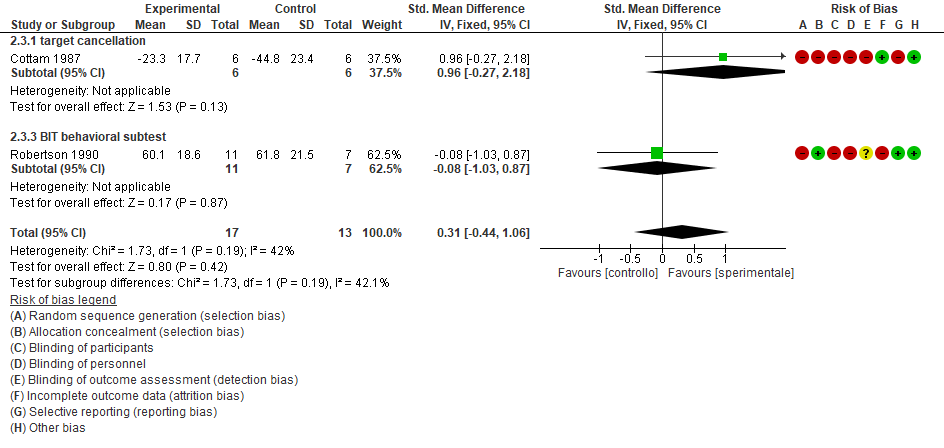


**Figure 12 - Functional improvement ADL immediate effects (at the end of treatment)**


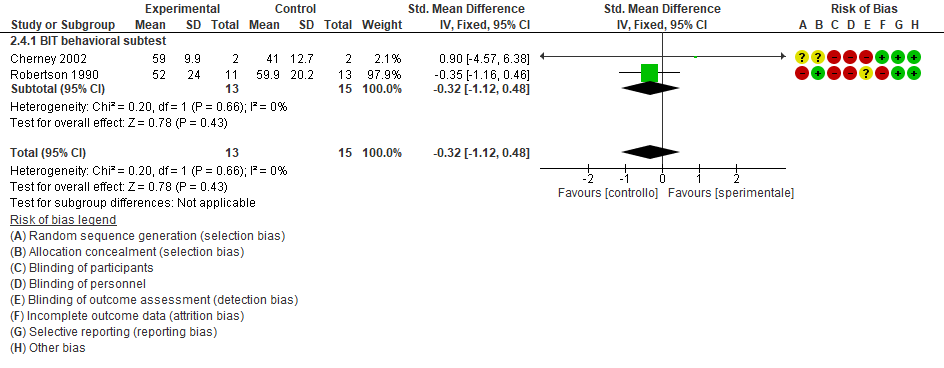


## **Evidence to Decision Framework**

| **PICO 2: In patients with unilateral spatial neglect after stroke, should visuospatial training be used compared with other interventions for improving functional outcomes?** | |
| --- | --- |
| **Population:** | Adults (>18 years) with post-stroke spatial hemineglect |
| **Intervention:** | Visual-spatial training |
| **Comparison:** | No treatment, other treatments |
| **Outcomes:** | Improved ADL functional persistent effects (at least 1 month after the end of treatment); Functional improvement ADL immediate effects (at the end of treatment); Improvement on neuropsychological tests - persistent effects; Improvement on neuropsychological tests immediate effects (all scales). |
| **Setting:** | Any setting |
| **Outlook:** | National Health Service (NHS) |
| **Conflicts of interest:** | No relevant or potentially relevant interest has been identified. All panel members present at the meeting voted, determining the direction and strength of the recommendation. |

**Rating**

| Problem  Is the problem a priority? | | |
| --- | --- | --- |
| Ratings | Searching for evidence | Additional considerations |
| ○ No  ○ Probably not  ○ Probably yes **● Yes**  **○** Varied  ○ Don't know | Neglect (or Unilateral Spatial Hemineglect) is a neuropsychological syndrome characterized by the inability of the patient to perceive or pay attention to objects, people, or representations, located in a visual hemifield (usually contralateral to the lesion), and to act on that side of the space (Kerkhoff et al., 2001; Husain, 2008). Neglect is usually associated with a lesion of the right hemisphere, especially in the lower part of the parietal lobe, and affects the left half of the personal or extra-personal space. Neglect simultaneously encompasses several factors: perception (hemi-inattention), action (motor negligence), and representation (cognitive functions). However, there can be several parts of space that can be affected by Neglect: personal space (space used by one's body); peri-personal space (space accessible by hand); extrapersonal space (space verifiable only by sight or hearing) (Spaccavento et al., 2017).  This disorder, whose symptoms are complex, brings with it a series of consequences that affect the performance of daily activities (dressing, personal hygiene), difficulty in reading, disorientation during walking, as well as reduced adherence to the proposed treatments, preventing the patient's general functional recovery. The presence of neglect is also associated with a longer hospital stay, a lower likelihood of returning to one's home after hospital discharge, and a greater risk of falls (Chen et al. 2015; Campbell et al., 2010; Bosma et al., 2020).  There is not a lot of data available on the occurrence of neglect. A recent systematic review (Esposito et al.,2021) estimated the prevalence of neglect after unilateral stroke from 20-40%, regardless of the type of lesion, the methodology followed for diagnosis, and the event of stroke.  Rehabilitation of neglect is essential to effectively restore the functions affected in this type of syndrome. In addition to this, the complexity of the symptoms of neglect makes it necessary to design specific rehabilitation interventions for the different existing types of this syndrome, in order to ensure the possibility that the patient achieves a certain degree of functional independence in daily activities (Li et al., 2015; Spaccavento et al., 2017).  Despite the wide spectrum of rehabilitation treatments currently used, it is not yet possible to formally recommend a rehabilitation technique. Among the proposed rehabilitation approaches, there is prismatic adaptation and visuo-exploratory training, but the available evidence is still uncertain, especially with regard to long-term clinical efficacy.  Several international guidelines (Winstein et al., 2016, NICE 2019; VA/DoD 2019) suggest the use of different rehabilitation treatments but do not provide guidance on which interventions should be considered first-line, second-line, and third-line.    **References**  - Bosma MS, Nijboer TWC, Caljouw MAA, Achterberg WP (2020) Impact of visuospatial neglect post-stroke on daily activities, participation and informal caregiver burden: A systematic review. Ann Phys Rehab Med 63(4):344-358.  - Campbell GB, Matthews JT (2010). An integrative review of factors associated with falls during post-stroke rehabilitation. J Nurs Scholarsh 42(4):395–404. <https://doi.org/10.1111/j.1547-5069.2010.01369.x>  - Chen P, Hreha K, Kong Y, Barrett AM (2015) Impact of spatial neglect in stroke rehabilitation: evidence from the setting of an inpatient rehabilitation facility. Arch Phys Med Rehabil 96(8):1458–1466. <https://doi.org/10.1016/j.apmr.2015.03.019>  -Esposito E, Shekhtman G, Chen P. (2021) Prevalence of spatial neglect post-stroke: A systematic review. Ann Phys Rehabil Med. 64(5):101459. <https://doi.org/10.1016/j.rehab.2020.10.010>  - Kerkhoff G (2001) Spatial hemineglect in humans. Prog Neurobiol 63(1):1-27. <https://doi.org/10.1016/s0301-0082(00)00028-9>  - Husain M. (2008) Hemispatial neglect. Handb Clin Neurol 88:359-372. <https://doi.org/10.1016/S0072-9752(07)88018-3>  - Li K, Malhotra PA (2015) Spatial neglect. Pract Neurol 15:333–339. <https://doi.org/10.1136/practneurol-2015-001115>  - Mazzucchi, A. (2016). La riabilitazione neuropsicologica. Premesse teoriche e applicazioni cliniche. Editore: Edra.  - Spaccavento S, Cellamare F, Falcone R, Loverre A, Nardulli R (2017) Effect of subtypes of neglect on functional outcome in stroke patients. Ann Phys Rehabil Med 60(6):376–381. <https://doi.org/10.1016/j.rehab.2017.07.245>  -The Management of Stroke Rehabilitation Work Group. VA/DoD CLINICAL PRACTICE GUIDELINE FOR THE MANAGEMENT OF STROKE REHABILITATION. Available at: <https://www.healthquality.va.gov/guidelines/Rehab/stroke/VADoDStrokeRehabCPGFinal8292019.pdf>  - National Clinical Guideline Centre (NICE). Stroke Rehabilitation in Adults. London: NICE, 2013. Aggiornamento 2019. Clinical guideline no. 162. Available at: <https://www.nice.org.uk/guidance/cg162> (Last access: 6 February 2023).  -Winstein CJ, Stein J, Arena R, Bates B, Cherney LR, Cramer SC, et al. (2016) Guidelines for adult stroke rehabilitation and recovery: a guideline for healthcare professionals from the American Heart Association/American Stroke Association. Stroke 47(6): e98–e169. <https://doi.org/10.1161/STR.0000000000000098> |  |
| **Desirable effects**  How considerable are the expected desirable effects? | | |
| Ratings | Searching for evidence | Additional considerations |
| ○ Irrelevant  ○ Small  ● **Moderate**  ○ Large  ○ Vary  ○ Don't know | Comparison**:** visuo-spatial training vs other treatments  **Source:** Longley et al., 2021, and subsequent updates   \| Outcomes \| Anticipated absolute effect^*^ (95% CI) \| \| Relative Effect (95% CI) \| No of participants(studies) \| Quality of Evidence (GRADE) \| \| --- \| --- \| --- \| --- \| --- \| --- \| \| Risk with other treatments \| Risk visual -spatial with training \| \| Improved ADL functional persistent effects (at least 1 month after the end of treatment). Outcome assessed with any scale/test. Comparison of training vs other active treatments \| Mean improved ADL functional effects persist (at least 1 month after the end of treatment). Outcome assessed with any scale/test. comparison of visual scanning vs other active treatments was 0 SD \| SMD 0.57 SD greater (0.31 greater a 1.45 greater) \| - \| 22 (2 RCT)^1,2^ \| ⨁◯◯◯ Very low ^a,b^ \| \| ADL functional improvement. Immediate effects. Comparison: Training vs Active Interventions Assessed With: Any Scale \| The average ADL functional improvement. Immediate effects. Comparison: Visual scanning vs active interventions was 0 SD \| SMD 0.24 SD Greater (0.48 less than 0.96 Greater) \| - \| 31 (2 RCT)^1,3^ \| ⨁◯◯◯ Very low ^a,b^ \| \| Improvement in neuropsychological tests. Persistent effects. Comparison: training vs control group assessed with: Any scale/test \| Average improvement in neuropsychological tests. Persistent effects. Comparison: Visual scanning vs control group was 0 \| SMD 0.31 Greater (0.44 less than 1.06 Greater) \| - \| 30 (2 RCT)^4,5^ \| ⨁◯◯◯ Very low ^a,d,e,f^ \| \| Improvement in neuropsychological tests. Immediate effects. Comparison: training vs control group evaluated with: BIT \| Average improvement in neuropsychological tests. Immediate effects. Comparison: Visual scanning vs control group was 0 \| SMD 0.32 less (1.12 Less Than 0.48 Greater) \| - \| 28 (2 RCT)^5,6^ \| ⨁◯◯◯ Very low ^a,c,f^ \|   a. Lowered by one level due to low sample size and low number of events.  b. Lowered by two levels due to methodological limitations: included studies are at risk of bias for sample selection, lack of blindness by patients and clinicians, loss to follow-up and selective publication of outcomes.  c. Lowered by two levels due to methodological limitations: the included studies are at high risk of bias in one study and at unclear risk in a second sample selection study; both studies are at high risk for lack of blindness by patients and clinicians. One study is at high risk of loss bias at follow-up.  d. Lowered by two levels due to methodological limitations: included studies are at high risk of bias for sample selection, lack of blindness by patients and clinicians in the study. One study is at high risk of loss bias at follow-up, and one study is at risk of selective publication of outcomes.  e. I2=42%  f. A study (Robertson et al., 1990) includes both left and right hemispheric stroke patients.  **References**  1. Luukkainen-Markkula R, Tarkka IM, Pitkänen K, Sivenius J, Hämäläinen H. (2009) Rehabilitation of hemispatial neglect: A randomized study using either arm activation or visual scanning training. Restor Neurol Neurosci 27(6):663-672. <https://10.3233/RNN-2009-0520>  2. Ferreira HP, Leite Lopes MA, Luiz RR, Cardoso L, André C. (2011) Is visual scanning better than mental practice in hemispatial neglect? Results from a pilot study. Top Stroke Rehabil 18(2):155-161. <https://10.1310/tsr1802-155>  3. Katz N, Ring H, Naveh Y, Kizony R, Feintuch U, Weiss PL (2005) Interactive virtual environment training for safe street crossing of right hemisphere stroke patients with unilateral spatial neglect. Disabil Rehabil 27(20):1235-1243. <https://10.1080/09638280500076079>  4. Cottam GL. Visual Scanning Training for Right Hemispheric Stroke Patients Exhibiting Hemispatial Neglect 1987 [Dissertation].  5. Robertson IH, Gray JM, Pentland B, Waite LJ. (1990) Microcomputer-based rehabilitation for unilateral left visual neglect: a randomized controlled trial. Arch Phys Med Rehabil 71(9):663-668.  6. Cherney LR, Halper AS, Papachronis D. (2003) Two approaches to treating unilateral neglect after right hemisphere stroke: a preliminary investigation. Top Stroke Rehabil 9(4):22-33. <https://10.1310/BWY3-7GQL-596Y-V17J>  **Other data**  The study conducted by Elshout et al. (2021) was not included in the meta-analysis. It reported that, in the group undergoing a "congruent movement training" intervention, there was a positive effect on neglect symptoms when assessed using three different tests after treatment. However, this effect was not observed in the group that received visual scanning training (VST). The study also highlights that the sample size was too small to accurately demonstrate any differences between the two groups. | The panel suggests the inclusion of an Italian study, Paolucci et al. (1996), not identified by the systematic search of the literature. The study, conducted on 20 patients diagnosed with neglect, evaluated with a battery of tests: Letter Cancellation Test, the Barrage Test, Sentence Reading Test and the Wundt-Jastrow Area Illusion test.  Patients were randomized to a visual-exploratory intervention for 8 weeks (immediate training) or no treatment (Delayed group).  At post-treatment, an improvement was observed in both groups in the cancellation test while, in the Sentence Reading Test, significant improvements were reported only for the group assigned to the "immediate" intervention: mean score 2.56 (SD = 2.83) before treatment, and 5.56 (SD = 1.01) post-treatment.  Also, at the Wundt-Jastrow Area Illusion test, greater pre- and post-differences were observed for the group assigned to the experimental intervention compared to the untreated group.  Both groups also showed improvement on the Semi-Structured Scale for the Functional Evaluation of Extrapersonal Neglect test post-treatment. The "immediate" group goes from a score of 9 (range 4-18) before training to 3 (range 0-13) after training.  - Antonucci G et al. (1995) Effectiveness of neglect rehabilitation in a randomised group study. J Clin Exp Neuropsychol 17(3):383-389.  - Paolucci et al. (1996) Facilitatory effect of neglect rehabilitation on the recovery of left hemiplegic stroke patients: a cross-over study. J Neurol 243:308-314. |
| **Undesirable effects**  How considerable are the expected undesirable effects? | | |
| Ratings | Searching for evidence | Additional considerations |
| **● Irrelevant**  ○ Small  ○ Moderate  ○ Large  ○ Vary  ○ Don't know | The studies identified in the literature did not identify adverse events due to treatment with visuo-spatial training |  |
| **Certainty of evidence**  What is the overall certainty of the evidence of efficacy and safety? | | |
| Ratings | Searching for evidence | Additional considerations |
| **● Very low**  **○** Low  ○ Moderate  ○ High  ○ No study included | The quality of the evidence was found to be very low for all the outcomes evaluated.  The quality was lowered mainly due to the risk of bias in RCTs (selection bias) and inaccuracy due to the very low sample size. |  |
| **Values**  Is there uncertainty or variability about how important people may consider the main outcomes? | | |
| Ratings | Searching for evidence | Additional considerations |
| ○ Important uncertainty  or variability  ○ Probably important uncertainty or variability  **● Probably not important uncertainty or variability**  ○ No major uncertainty or variability | No studies have been identified that have reported data on the value that people with neglect place on rehabilitation treatment outcomes. | In the absence of available studies, the panel believes that the outcomes considered in the research are shared by patients and their families. |
| Balance of effects  Does the balance between desirable and undesirable effects favor intervention or comparison? | | |
| Ratings | Searching for evidence | Additional considerations |
| ○ Promotes comparison  ○ Likely favors comparison  ○ Favors neither comparison nor treatment  ○ Probably favors treatment **● Favors treatment**  ○ Varies  ○ Don't know | Considering the absence of undesirable effects, the balance between benefits and risks favors intervention |  |
| Resources required  How much are the resources required (costs)? | | |
| Ratings | Searching for evidence | Additional considerations |
| ○ Very high costs  **● Moderate costs**  ○ Irrelevant costs and savings  ○ Moderate savings  ○ High savings  ○ Varies  ○ Don't know | Economic Evaluation Report |  |
| Quality of evidence in relation to the resources needed  What is the quality of the evidence in relation to the resources needed (costs)? | | |
| Ratings | Searching for evidence | Additional considerations |
| ○ Very low  ○ Low  ○ Moderate  ○ High  ● **No study included** | Economic Evaluation Report |  |
| Cost-effectiveness  Does cost-effectiveness analysis favour intervention or comparison? | | |
| Giudizi | Ricerca delle prove | Considerazioni aggiuntive |
| ○ Promotes comparison  ○ Likely favors comparison  ○ Favors neither comparison nor treatment  ○ Probably favors treatment  ○ Favors treatment  ○ Varies  ● **Don't know** | Economic Evaluation Report |  |
| **Equity**  What would be the impact in terms of equity? | | |
| Ratings | Searching for evidence | Additional considerations |
| ○ Reduces equity  ○ Likely reduces equity  ○ Probably no impact  **●Likely improves equity**  ○ Improves equity  ○ Varies  ○ I don't know | No studies have been identified that have reported data on the impact of rehabilitation treatment with prismatic lenses on equity. | See the data on the survey on access to services available in Italy. |
| **Acceptability**  Is the intervention acceptable for the main stakeholders? | | |
| Ratings | Searching for evidence | Additional considerations |
| ○ No  ○ Probably not  **● Probably yes**  ○ Yes  ○ Varies  ○ I don't know | Literature search did not identify any studies. | The panel believes that the visual-spatial training intervention does not pose problems of acceptability by the different stakeholders |
| Feasibility  Is the implementation of the intervention feasible? | | |
| Ratings | Searching for evidence | Additional considerations |
| ○ No  ○ Probably not  ○ Probably yes  **● Yes**  ○ Varies  ○ I don't know | The literature search led to the identification of two studies, described narratively below.  **Chen et al. (2017)** reported the results of an online survey aimed at healthcare professionals to learn about their knowledge, opinions and obstacles on rehabilitation treatments for patients with neglect. In particular, a clinical case was presented to the professionals, and they were asked to plan a treatment plan in different post-stroke phases, assuming an ideal scenario and one adapted to clinical practice. One hundred and twenty-seven professionals from various medical disciplines in 23 countries responded to the survey. Based on years of experience, the professionals were divided into two subgroups: a subgroup consisting of 30 highly specialized experts with at least 10 years of clinical or teaching experience and at least five scientific publications on neglect; a second subgroup of 97 experts with less experience. Among the treatments chosen by the experts, visual scanning, "Active limb activation", prismatic adaptation and "sustained attention training" were the first five selected in the conditions of an ideal scenario. In both scenarios, more treatments were chosen in the acute (2 weeks to 3 months) and subacute (3 to 12 months post-stroke) phase, compared to the early (<2 weeks post-stroke) or chronic (>12 months post-trauma) phases.  As for the obstacles perceived by the professionals involved in the survey, 16.3% of professionals stated that they had no obstacles, and 15.8% did not consider spatial neglect a priority. About 44.6% of participants reported that they did not have the necessary time to treat patients with neglect and 37.6% that they did not have the necessary instrumentation. Other answers concerned the difficulty of interdisciplinary relationships, the absence of standardized protocols, and the lack of effectiveness of treatments.  **Evald et al. (2020)** reported the results of an online survey conducted in Denmark aimed at healthcare professionals on the diagnosis and treatment of neglect. Among 525 professionals involved in the survey, 411 (78.3%) reported that, in their clinical practice, treatments for neglect were usually delivered. A deeper analysis of the responses showed that a small proportion of practitioners (n = 78, 14.9%) reported that no neglect treatments were offered in their clinical setting, and a smaller number (n = 36, 6.9%) were unaware of the availability of these treatments.  With regard to the professional figures involved, occupational therapists were among the most involved, followed by physiotherapists, especially in public health services. About a third of respondents reported the figure of nurses and social workers, more frequent in the hospital setting. Less involved were psychologists, speech therapists and doctors.  With regard to treatment initiation, participants employed in a hospital setting reported timely initiation of treatment for neglect (within one month of the event) compared to that reported by professionals employed in the private sector (3 to 6 months post-event).  The frequency of treatment was also higher in the hospital and private setting (once a day) than in the municipal setting (2-3 times a week). The duration of the sessions reported was 21-30 minutes for the hospital and private settings and 31-40 minutes for the municipal setting.  The majority of participants reported that personal experience and contact with colleagues were the basis for their choice of treatment. Few practitioners have reported using national guidelines or other studies as a source of evidence when choosing the type of treatment.  **References**   1. Chen P, Pitteri M, Gillen G, Ayyala H. (2018) Ask the experts how to treat individuals with spatial neglect: a survey study. Disabil Rehabil. 40(22):2677-2691. <https://doi.org/10.1080/09638288.2017.1347720> 2. Evald L, Wilms IL, Nordfang M. (2020) Treatment of spatial neglect in clinical practice: A nationwide survey. Acta Neurol Scand. Jan;141(1):81-89. <https://doi.org/10.1111/ane.13179> |  |

**Summary of judgments**

|  | **Ratings** | | | | | | |
| --- | --- | --- | --- | --- | --- | --- | --- |
| **Priority of the Problem** | No | Probably not | Probably yes | **Yes** |  | Varies | Don't know |
| **Desirable effects** | Irrelevant | Small | **Moderate** | Large |  | Varies | Don't know |
| **Undesirable effects** | Large | Moderate | Small | **Irrelevant** |  | Varies | Don't know |
| **Quality of Evidence** | **Very low** | Low | Moderate | High |  |  | No studies included |
| **Values** | Important uncertainty or variability | Probably important uncertainty or variability | **Probably not important uncertainty or variability** | No major uncertainty or variability |  |  |  |
| **Balance of effects** | In favor of confrontation | Probably in favor of comparison | It does not favor either discussion or intervention | Probably in favor of the intervention | **In favor of intervention** | Varies | Don't know |
| **Resources Needed** | Large costs | **Moderate costs** | Negligible costs and savings | Moderate savings | Large savings | Varies | Don't know |
| **Quality of evidence in relation to the resources needed** | Very low | Low | Moderate | High |  |  | **No studies included** |
| **Cost Effectiveness** | In favor of confrontation | Probably in favor of comparison | Not favor either comparison or treatment | Probably in favor of the intervention | In favor of intervention | Varies | **No studies included** |
| **Equity** | Reduced | Probably reduced | Probably no impact | **Probably increased** | Increased | Varies | Don't know |
| **Acceptability** | No | Probably not | **Probably yes** | Yes |  | Varies | Don't know |
| **Feasibility** | No | Probably not | Probably yes | **Yes** |  | Varies | Don't know |

**Type of recommendation**

| Strong recommendation against intervention | Conditional recommendation against intervention | Conditional recommendation in favor of both intervention and discussion | **Conditional recommendation in favor of intervention** | Strong recommendation in favor of intervention |
| --- | --- | --- | --- | --- |
| ○ | ○ | ○ |  | ○ |

**Conclusion**

| Recommendations |
| --- |
| Recommendation (PICO 2 – Conditional recommendation): In patients with peri-personal neglect after stroke, it is indicated to use visuospatial training as part of the rehabilitation program (Certainty of evidence: very low). |
|  |
| Justification |
| The relatively low cost, the absence of side effects and the evidence in favor of the treatment, although of very low quality, justify, according to the panel, the proposal of this method for the treatment of neglect. |

| Considerations for subgroups |
| --- |
| Currently, it is not possible to draw reliable considerations for the specific treatment of individual subgroups of patients with neglect. |
| Implementation considerations |
| The training of health personnel is an important factor in the effectiveness of the intervention. It is suggested to provide training for dedicated staff. |

| Monitoring and evaluation |
| --- |
|  |
| Research priorities |
| It would be desirable to conduct multicenter, comparative studies, conducted in patients in the acute and chronic phases, at different post-treatment follow-ups (>6 months). Studies should evaluate clinical outcomes such as the number of falls, length of stay, and quality of life.  It would also be desirable, in conducting comparative studies, to define the types and severity of neglect.  Finally, the panel suggests conducting qualitative studies on patients with neglect and caregivers to explore the acceptability of rehabilitation treatments |

**Table S12 Table of evidence (GRADE)**

**Comparison:** Visual-spatial training method compared to other treatments for patients with spatial hemineglect

**Setting:** Any

**Reference:** Longley et al. (2021)

| **Certainty assessment** | | | | | | | **No of patients** | | **Effect** | | **Quality of evidence**  **Study design** | **Importance**  **Risk of distortion** |
| --- | --- | --- | --- | --- | --- | --- | --- | --- | --- | --- | --- | --- |
| **No of studies** | **Study design** | **Risk of distortion** | **Lack of reproducibility of results** | **Lack of generalizability** | **No of studies** | **Study design** | **Risk of distortion** | **Lack of reproducibility of results** | **Lack of generalizability** | **No of studies** |  |  |
| **Improved ADL functional persistent effects (at least 1 month after the end of treatment). Outcome assessed with any scale/test. Comparison of visuo-spatial training vs other active treatments** | | | | | | | | | | | | |
| 2^1,2^ | Randomized trials | very serious ^a^ | not important | not important | Serious ^b^ | none | 11 | 11 | - | SMD **0.57 SD Greater**  (0.31 Greater to 1.45 Greater) | ⨁◯◯◯  Very low | critical |
| **ADL functional improvement. Immediate effects. Comparison: Visual scanning vs active interventions (assessed with: Any scale)** | | | | | | | | | | | | |
| 2^1,3^ | Randomized trials | very serious ^a^ | not important | not important | Serious ^b^ | none | 14 | 17 | - | SMD **0.24 SD Greater**  (0.48 less than 0.96 Greater) | ⨁◯◯◯  Very low | critical |
| **Improvement in neuropsychological tests. Persistent effects. Comparison: Visual scanning vs control group (assessed with: Any scale/test)** | | | | | | | | | | | | |
| 2^4,5^ | Randomized trials | very serious ^d^ | Serious ^e^ | Serious ^f^ | Serious ^b^ | none | 17 | 13 | - | SMD **0.31 Greater**  (0.44 less than 1.06 Greater) | ⨁◯◯◯  Very low | critical |
| **Improvement in neuropsychological tests. Immediate effects. Comparison: visual scanning vs control group (assessed with: BIT)** | | | | | | | | | | | | |
| 2^5,6^ | Randomized trials | very serious ^c^ | not important | Serious ^f^ | Serious ^b^ | none | 13 | 15 | - | SMD **0.32 Lower**  (1.12 Less Than 0.48 Greater) | ⨁◯◯◯Very low | critical |

**CI:** Confidence interval; **SMD:** Standardized mean difference

*Explanations*

a. Lowered by two levels due to methodological limitations: included studies are at risk of bias for sample selection, lack of blindness by patients and clinicians, loss to follow-up and selective publication of outcomes.

b. Lowered by one level due to low sample size and low number of events.

c. Lowered by two levels due to methodological limitations: the included studies are at high risk of bias in one study and at unclear risk in a second sample selection study; both studies are at high risk for lack of blindness by patients and clinicians. One study is at high risk of loss bias at follow-up.

d. Lowered by two notches due to methodological limitations: Included studies are at high risk of bias for sample selection, lack of blindness by patients and clinicians in a study. One study is at high risk of loss bias at follow-up and one study by selective publication of outcomes.

e. I2=42%

f. One study (Robertson 1990) includes both left and right hemispheric stroke patients.

**References**

1. Luukkainen-Markkula R, Tarkka IM, Pitkänen K, Sivenius J, Hämäläinen H. (2009) Rehabilitation of hemispatial neglect: A randomized study using either arm activation or visual scanning training. Restor Neurol Neurosci 27(6):663-672. <https://10.3233/RNN-2009-0520>

2. Ferreira HP, Leite Lopes MA, Luiz RR, Cardoso L, André C. (2011) Is visual scanning better than mental practice in hemispatial neglect? Results from a pilot study. Top Stroke Rehabil 18(2):155-161. <https://10.1310/tsr1802-155>

3. Katz N, Ring H, Naveh Y, Kizony R, Feintuch U, Weiss PL (2005) Interactive virtual environment training for safe street crossing of right hemisphere stroke patients with unilateral spatial neglect. Disabil Rehabil 27(20):1235-1243. <https://10.1080/09638280500076079>

4. Cottam GL. Visual Scanning Training for Right Hemispheric Stroke Patients Exhibiting Hemispatial Neglect 1987 [Dissertation].

5. Robertson IH, Gray JM, Pentland B, Waite LJ. (1990) Microcomputer-based rehabilitation for unilateral left visual neglect: a randomized controlled trial. Arch Phys Med Rehabil. 71(9):663-668.

6. Cherney LR, Halper AS, Papachronis D. (2003) Two approaches to treating unilateral neglect after right hemisphere stroke: a preliminary investigation. Top Stroke Rehabil. 9(4):22-33. <https://10.1310/BWY3-7GQL-596Y-V17J>

PICO 3: In patients with peri-personal neglect after stroke who are candidates for rehabilitation, when should prism adaptation or visuospatial training be initiated to optimize functional outcomes?**Table S13. Summary of the protocol**

| **Population:** | Adults over 18 years of age affected by right hemispheric stroke and peripersonal neglect (i.e., with difficulty in exploring the space whose boundary is defined by the extension of the upper limb-reaching, grasping), with both egocentric and allocentric forms. |
| --- | --- |
| **Intervention** | Prismatic lenses or visuo-spatial training |
| **Comparison** | No treatment, other non-pharmacological treatments |
| **Outcomes**: | Critical outcomes: Functional improvement measured with CBS, FMI and BI, Improvement on neuropsychological tests (line bisection test, cancellation/barrage, reading), Length of stay, Quality of life measured with any validated scale.  Important: Mood measured with rating scales (Beck Depression scale, HAD scale), Number of falls to the ground measured as the number of people with fractures, Fate at discharge.  *Other outcomes:* Acceptability (dropout, adherence to treatment, satisfaction with treatment), Feasibility, Values, Fairness. |
| **Setting** | Any setting |
| **Study design** | Systematic reviews of randomized controlled trials. Individual randomized trials  In the absence of RCT studies, observational studies with a control group. No case series and individual cases  Data sources and research strategy: See clinical questions on rehabilitation treatment |

**Figure 5 Study selection process**

**Identification of studies through databases**

Records removed prior to screening:

Duplicates removed

(n =1160)

Records identified through databases: Cochrane Library, Medline ed Embase (n = 2955)

**Identification**

Excluded records based on title and abstract = 1795

Records to be evaluated

(n = 1795)

**Screening**

Records to be evaluated as full text (n = 0)

Full text evaluated for eligibility (n = 0)

**Eligibility**

Inclusion studies (n = 0)

**Inclusion**

PICO 4: In stroke survivors with peri-personal neglect, are neglect-specific scales (e.g., CBS–KF-NAP, Zoccolotti scale) more accurate than non-specific ADL measures (e.g., Barthel Index, FIM) for evaluating functional status?

**Table S14. Summary of the protocol**

| **Population:** | Adults over 18 years of age affected by right hemispheric stroke and peripersonal neglect (i.e., with difficulty in exploring the space whose boundary is defined by the extension of the upper limb-reaching, grasping), with both egocentric and allocentric forms. |
| --- | --- |
| **Intervention** | Barthel Index or Functional Independence Measure |
| **Comparison** | Barthel Index or Functional Independence Measure |
| **Outcomes**: | Sensitivity and Specificity, Inter-operative validity, Test-retest validity |
| **Setting** | Any setting |
| **Study design** | Systematic reviews of randomized controlled trials. Individual randomized trials  In the absence of RCT studies, observational studies with a control group. No case series and individual cases  Data sources and research strategy: See clinical questions on rehabilitation treatment |

**Search strategy**

Cochrane Library (issue 01, 2023)

#1 [mh ^"cerebrovascular disorders"] or [mh "basal ganglia cerebrovascular disease"] or [mh "brain ischemia"] or [mh "carotid artery diseases"] or [mh "intracranial arterial diseases"] or [mh "intracranial embolism and thrombosis"] or [mh "intracranial hemorrhages"] or [mh ^stroke] or [mh "brain infarction"] or [mh ^"stroke, lacunar"] or [mh ^"vasospasm, intracranial"] or [mh ^"vertebral artery dissection"]

#2 (stroke or poststroke or "post‐stroke" or cerebrovasc* or brain next vasc* or cerebral next vasc* or cva* or apoplexy* or SAH):ti,ab

#3 ((brain* or cerebral* or cerebell* or intracran* or intracerebral) near/5 (isch*emi* or infarct* or thrombo* or emboli* or occlus*)):ti,ab

#4 ((brain* or cerebral* or cerebell* or intracerebral or intracranial or subarachnoid) near/5 (haemorrhage* or hemorrhage* or haematoma* or hematoma* or bleed*)):ti,ab 8129

#5 [mh ^hemiplegia] or [mh paresis]

#6 (hemipleg* or hemipar* or paresis or paretic):ti,ab

#7 #1 or #2 or #3 or #4 or #5 or #6

#8 [mh ^"perceptual disorders"] or [mh ^perception] or [mh "visual perception"] or [mh ^"space perception"] or [mh ^attention] or [mh ^"functional laterality"] or [mh ^"extinction, psychological"]

#9 (hemineglect or hemi‐neglect):ti,ab

#10 ((unilateral or spatial or hemispatial or hemi‐spatial or visual) near/5 neglect):ti,ab

#11 (inattention or hemi‐inattention or extinction):ti,ab

#12 ((perceptual or perception or visuospatial or visuo‐spatial or visuoperceptual or visuo‐perceptual or attention*) near/5 (disorder* or deficit* or impairment* or abilit* or problem*)):ti,ab

#13 #8 or #9 or #10 or #11 or #12

#14 #7 AND #13

#15 MeSH descriptor: [Sensitivity and Specificity] explode all trees

#16 ((Psychometric NEXT properties) or specificity or sensitivity or reliab* or valid* or clinimetric or diagnostic accuracy):ti,ab,kw

#17 #15 OR #16

#18 #14 AND #17 in Cochrane Review / Review Type Diagnostic 1

#19 #14 AND #17 in Trials

**Ovid MEDLINE(R) ALL <1946 to January 2023>**

1 cerebrovascular disorders/ or exp basal ganglia cerebrovascular disease/ or exp brain ischemia/ or exp carotid artery diseases/ or exp intracranial arterial diseases/ or exp "intracranial embolism and thrombosis"/ or exp intracranial hemorrhages/ or stroke/ or exp brain infarction/ or stroke, lacunar/ or vasospasm, intracranial/ or vertebral artery dissection/

2 (stroke or poststroke or post-stroke or cerebrovasc$ or brain vasc$ or cerebral vasc$ or cva$ or apoplex$ or SAH).tw.

3 ((brain$ or cerebr$ or cerebell$ or intracerebral or intracranial or subarachnoid) adj5 (haemorrhage$ or hemorrhage$ or haematoma$ or hematoma$ or bleed$)).tw.

4 ((brain$ or cerebr$ or cerebell$ or intracran$ or intracerebral) adj5 (isch?emi$ or infarct$ or thrombo$ or emboli$ or occlus$)).tw.

5 hemiplegia/ or exp paresis/

6 (hemipleg$ or hemipar$ or paresis or paretic).tw.

7 1 or 2 or 3 or 4 or 5 or 6

8 (hemineglect or hemi-neglect).tw.

9 perceptual disorders/ or perception/ or exp visual perception/ or space perception/ or attention/ or functional laterality/ or extinction, psychological/

10 ((unilateral or spatial or hemispatial or visual) adj5 neglect).tw.

11 (perception or inattention or hemi-inattention or extinction).tw.

12 ((perceptual or visuo?spatial or visuo?perceptual or attention$) adj5 (disorder$ or deficit$ or impairment$ or abilit$ or problem$)).tw.

13 8 or 9 or 10 or 11 or 12

14 (test or clinical measure* or assessment* or evaluation* or measurement* or screening tool* or outcome measure* or scale or instrument).tw.

15 (battery or checklist or checklists or subtest or subtests).tw.

16 (line adj2 (bisection or quadrisection or extension)).tw.

17 barrage.tw.

18 ((apples or circle or computerised or ellipses or haptics or hearts or letter or line or shape or star or symbol or word) adj2 cancellation).tw.

19 ((cancellation or detection or bisection) adj3 task*).tw.

20 (Mesulam or Schenkenberg).tw.

21 Judd stimuli.tw.

22 Cognitive Screen.tw.

23 (Kerkhoff or Visual search board or VSB or Kimura).tw.

24 ((article or indented paragraph or Munich or number or sentence or text or word) adj2 reading).tw.

25 (reading adj2 task*).tw.

26 (Vallar or Max 9pts).tw. or (WR or EWR).ti.

27 14 or 15 or 16 or 17 or 18 or 19 or 20 or 21 or 22 or 23 or 24 or 25 or 26

28 "Sensitivity and Specificity"/

29 (Psychometric properties or specificity or sensitivity or reliab* or valid* or clinimetric or diagnostic accuracy).mp.

30 28 or 29

31 7 and 13 and 27 and 30

32 animals/ not humans/

33 31 not 32

**Embase <1974 to 2023 January>**

1 stroke/ or cerebrovascular disease/ or exp basal ganglion hemorrhage/ or exp brain hematoma/ or exp brain hemorrhage/ or exp brain infarction/ or exp brain ischemia/ or exp carotid artery disease/ or cerebral artery disease/ or exp cerebrovascular accident/ or exp intracranial aneurysm/ or exp occlusive cerebrovascular disease/ or stroke patient/ or stroke unit/

2 (stroke or poststroke or post-stroke or cerebrovasc$ or brain vasc$ or cerebral vasc$ or cva$ or apoplex$ or SAH).tw.

3 ((brain$ or cerebr$ or cerebell$ or intracran$ or intracerebral) adj5 (isch?emi$ or infarct$ or thrombo$ or emboli$ or occlus$)).tw.

4 ((brain$ or cerebr$ or cerebell$ or intracerebral or intracranial or subarachnoid) adj5 (haemorrhage$ or hemorrhage$ or haematoma$ or hematoma$ or bleed$)).tw.

5 hemiparesis/ or hemiplegia/ or paresis/

6 (hemipleg$ or hemipar$ or paresis or paretic).tw.

7 1 or 2 or 3 or 4 or 5 or 6

8 exp *perception disorder/ or exp *perception/ or exp *attention/ or *attention disturbance/ or *visual deprivation/ or *neglect/ or *hemispatial neglect/ or *"unilateral neglect syndrome"/

9 (hemineglect or hemi-neglect).tw.

10 ((unilateral or spatial or hemi?spatial or visual) adj5 neglect).tw.

11 (inattention or hemi-inattention or extinction).tw.

12 8 or 9 or 10 or 11

13 (test or clinical measure* or assessment* or evaluation* or measurement* or screening tool* or outcome measure* or scale or instrument).tw.

14 (battery or checklist or checklists or subtest or subtests).tw.

15 (line adj2 (bisection or quadrisection or extension)).tw.

16 (line adj2 (bisection or quadrisection or extension)).tw.

17 ((apples or circle or computerised or ellipses or haptics or hearts or letter or line or shape or star or symbol or word) adj2 cancellation).tw.

18 ((cancellation or detection or bisection) adj3 task*).tw.

19 (Mesulam or Schenkenberg).tw.

20 Judd stimuli.tw.

21 Cognitive Screen.tw.

22 (Kerkhoff or Visual search board or VSB or Kimura).tw.

23 ((article or indented paragraph or Munich or number or sentence or text or word) adj2 reading).tw.

24 (reading adj2 task*).tw.

25 (Vallar or Max 9pts).tw. or (WR or EWR).ti.

26 or/13-25

27 (Psychometric properties or specificity or sensitivity or reliab* or valid* or clinimetric or accuracy).mp.

28 diagnostic accuracy/

29 27 or 28

30 7 and 12 and 26 and 29

**APA PsycInfo <1806 to January Week 1 2023>**

1 cerebrovascular disorders/ or cerebral hemorrhage/ or exp cerebral ischemia/ or cerebrovascular accidents/ or subarachnoid hemorrhage/

2 (stroke or poststroke or post-stroke or cerebrovasc$ or brain vasc$ or cerebral vasc$ or cva$ or apoplex$ or SAH).tw.

3 ((brain$ or cerebr$ or cerebell$ or intracran$ or intracerebral) adj5 (isch?emi$ or infarct$ or thrombo$ or emboli$ or occlus$)).tw.

4 ((brain$ or cerebr$ or cerebell$ or intracerebral or intracranial or subarachnoid) adj5 (haemorrhage$ or hemorrhage$ or haematoma$ or hematoma$ or bleed$)).tw.

5 hemiparesis/ or hemiplegia/

6 (hemipleg$ or hemipar$ or paresis or paretic).tw.

7 or/1-6

8 sensory neglect/

9 exp perceptual disturbances/

10 exp perception/

11 exp attention/

12 "extinction (learning)"/

13 (hemineglect or hemi-neglect).tw.

14 ((unilateral or spatial or hemispatial or visual) adj5 neglect).tw.

15 (inattention or hemi-inattention or extinction).tw.

16 ((perceptual or perception or visuo?spatial or visuo?perceptual or attention$) adj5 (disorder$ or deficit$ or impairment$ or abilit$)).tw.

17 or/8-16

18 (test or clinical measure* or assessment* or evaluation* or measurement* or screening tool* or outcome measure* or scale or instrument).tw.

19 (battery or checklist or checklists or subtest or subtests).tw.

20 (line adj2 (bisection or quadrisection or extension)).tw.

21 ((apples or circle or computerised or ellipses or haptics or hearts or letter or line or shape or star or symbol or word) adj2 cancellation).tw.

22 ((cancellation or detection or bisection) adj3 task*).tw.

23 (Mesulam or Schenkenberg).tw.

24 Cognitive Screen.tw.

25 (Kerkhoff or Visual search board or VSB or Kimura).tw.

26 ((article or indented paragraph or Munich or number or sentence or text or word) adj2 reading).tw.

27 (reading adj2 task*).tw.

28 (Vallar or Max 9pts).tw. or (WR or EWR).ti.

29 or/18-28

30 exp Test Sensitivity/

31 exp Test Reliability/

32 (Psychometric properties or specificity or sensitivity or reliab* or valid* or clinimetric or accuracy).mp.

33 30 or 31 or 32

34 7 and 17 and 29 and 33

**CINAHL EBSCOHOST**

(2023 January)

S43 S26 AND S39 AND S42

S42 S40 OR S41

S41 TX ("Psychometric properties" OR specificity OR sensitivity OR reliab* OR valid* OR clinimetric OR "diagnostic accuracy")

S40 (MH "Sensitivity and Specificity")

S39 S27 OR S28 OR S29 OR S30 OR S31 OR S32 OR S33 OR S34 OR S35 OR S36 OR S37 OR S38

S38 ((TI Vallar OR AB Vallar) OR (TI "Max 9pts" OR AB "Max 9pts")) OR (TI WR OR TI EWR)

S37 ((TI reading OR AB reading) N2 (TI task* OR AB task*))

S36 (((TI article OR AB article) OR (TI "indented paragraph" OR AB "indented paragraph") OR (TI Munich OR AB Munich) OR (TI number OR AB number) OR (TI sentence OR AB sentence) OR (TI text OR AB text) OR (TI word OR AB word)) N2 (TI reading OR AB reading))

S35 ((TI Kerkhoff OR AB Kerkhoff) OR (TI "Visual search board" OR AB "Visual search board") OR (TI VSB OR AB VSB) OR (TI Kimura OR AB Kimura))

S34 (TI "Cognitive Screen" OR AB "Cognitive Screen")

S33 ((TI Mesulam OR AB Mesulam) OR (TI Schenkenberg OR AB Schenkenberg))

S32 (((TI cancellation OR AB cancellation) OR (TI detection OR AB detection) OR (TI bisection OR AB bisection)) N3 (TI task* OR AB task*))

S31 (((TI apples OR AB apples) OR (TI circle OR AB circle) OR (TI computerised OR AB computerised) OR (TI ellipses OR AB ellipses) OR (TI haptics OR AB haptics) OR (TI hearts OR AB hearts) OR (TI letter OR AB letter) OR (TI line OR AB line) OR (TI shape OR AB shape) OR (TI star OR AB star) OR (TI symbol OR AB symbol) OR (TI word OR AB word)) N2 (TI cancellation OR AB cancellation))

S30 (TI barrage OR AB barrage)

S29 ((TI line OR AB line) N2 ((TI bisection OR AB bisection) OR (TI quadrisection OR AB quadrisection) OR (TI extension OR AB extension)))

S28 ((TI battery OR AB battery) OR (TI checklist OR AB checklist) OR (TI checklists OR AB checklists) OR (TI subtest OR AB subtest) OR (TI subtests OR AB subtests))

S27 ((TI test OR AB test) OR (TI "clinical measure*" OR AB "clinical measure*") OR (TI assessment* OR AB assessment*) OR (TI evaluation* OR AB evaluation*) OR (TI measurement* OR AB measurement*) OR (TI "screening tool*" OR AB "screening tool*") OR (TI "outcome measure*" OR AB "outcome measure*") OR (TI scale OR AB scale) OR (TI instrument OR AB instrument))

S26 S12 AND S25

S25 S13 OR S14 OR S15 OR S16 OR S17 OR S20 OR S21 OR S24

S24 S22 AND S23

S23 TI (disorder* or deficit* or impairment* or abilit*) or AB (disorder* or deficit* or impairment* or abilit*)

S22 TI (perceptual or perception or visuo#spatial or visuo#perceptual or attention*) or AB (perceptual or perception or visuo#spatial or visuo#perceptual or attention*)

S21 TI (inattention or hemi‐inattention or extinction) or AB (inattention or hemi‐inattention or extinction)

S20 S18 AND S19

S19 TI (neglect) or AB (neglect)

S18 TI (unilateral or spatial or hemi#spatial or visual) or AB (unilateral or spatial or hemi#spatial or visual)

S17 TI (hemineglect or hemi‐neglect) or AB (hemineglect or hemi‐neglect)

S16 (MH "attention")

S15 (MH "Perception+")

S14 (MH "Perceptual Disorders+")

S13 (MH "Unilateral Neglect") OR (MH "Unilateral Neglect (Saba CCC)") OR (MH "Unilateral Neglect (NANDA)")

S12 S1 or S2 or S3 or S6 or S9 or S10 or S11

S11 TI ( hemipleg* or hemipar* or paresis or paretic ) or AB ( hemipleg* or hemipar* or paresis or paretic )

S10 (MH "Hemiplegia")

S9 S7 AND S8

S8 TI ( haemorrhage* or hemorrhage* or haematoma* or hematoma* or bleed* ) or AB ( haemorrhage* or hemorrhage* or haematoma* or hematoma* or bleed* )

S7 TI ( brain* or cerebr* or cerebell* or intracerebral or intracranial or subarachnoid ) or AB ( brain* or cerebr* or cerebell* or intracerebral or intracranial or subarachnoid )

S6 S4 AND S5

S5 TI ( ischemi* or ischaemi* or infarct* or thrombo* or emboli* or occlus* ) or AB ( ischemi* or ischaemi* or infarct* or thrombo* or emboli* or occlus* )

S4 TI ( brain* or cerebr* or cerebell* or intracran* or intracerebral ) or AB ( brain* or cerebr* or cerebell* or intracran* or intracerebral )

S3 TI ( stroke or poststroke or post‐stroke or cerebrovasc* or brain vasc* or cerebral vasc or cva or apoplex or SAH ) or AB ( stroke or poststroke or post‐stroke or cerebrovasc* or brain vasc* or cerebral vasc or cva or apoplex or SAH )

S2 (MH "Stroke Patients") OR (MH "Stroke Units")

S1 (MH "Cerebrovascular Disorders") OR (MH "Basal Ganglia Cerebrovascular Disease+") OR (MH "Carotid Artery Diseases+") OR (MH "Cerebral Ischemia+") OR (MH "Cerebral Vasospasm") OR (MH "Intracranial Arterial Diseases+") OR (MH "Intracranial Embolism and Thrombosis") OR (MH "Intracranial Hemorrhage+") OR (MH "Stroke") OR (MH "Vertebral Artery Dissections")

**Figure 6 Study selection process**

**Identification of studies through databases**

Records removed prior to screening:

Duplicates removed

(n = 860)

Records identified through databases: Cochrane Library, Medline ed Embase (n = 3126)

**Identification**

Excluded records based on title and abstract = 2295

Records to be evaluated

(n =2300)

**Screening**

Records to be evaluated as full text (n=5)

Full text excluded with reason for exclusion: (n = 5)

Full text evaluated for eligibility (n = 5)

**Eligibility**

Inclusion studies = 0

**Inclusion**

**Table S15 Excluded studies**

| **References** | **Reason for exclusion** |
| --- | --- |
| Chen P, Chen CC, Hreha K, Goedert KM, Barrett AM (2015) Kessler Foundation Neglect Assessment Process uniquely measures spatial neglect during activities of daily living. Arch Phys Med Rehabil 96(5):869-876. <https://10.1016/j.apmr.2014.10.023> | Irrelevant outcomes |
| Chen P, Hreha K, Kong Y, Barrett AM. (2015) Impact of spatial neglect on stroke rehabilitation: evidence from the setting of an inpatient rehabilitation facility. Arch Phys Med Rehabil 96(8): 1458–1466. <https://doi.org/10.1016/j.apmr.2015.03.019> | Double publication of Chen 2015 |
| Pitteri M, Chen P, Passarini L, Albanese S, Meneghello F, Barrett AM. (2018) Conventional and functional assessment of spatial neglect: Clinical practice suggestions. Neuropsychology 32(7):835-842. <https://10.1037/neu0000469> | Irrelevant outcomes |
| Marques CLS, de Souza JT, Gonçalves MG, da Silva TR, da Costa RDM, Modolo GP, Corrente JE, Bazan R, Luvizutto GJ (2019) Validation of the Catherine Bergego Scale in patients with unilateral spatial neglect after stroke. Dement Neuropsychol 13(1):82-88. <https://10.1590/1980-57642018dn13-010009> | CBS Portuguese language adaptation |
| Nishida D, Mizuno K, Tahara M, Shindo S, Watanabe Y, Ebata H, Tsuji T. (2021) Behavioral assessment of unilateral spatial neglect with the Catherine Bergego Scale (CBS) using the Kessler Foundation Neglect Assessment Process (KF-NAP) in patients with subacute stroke during rehabilitation in Japan. Behav Neurol. 8825192. <https://10.1155/2021/8825192> | Japanese language adaptation by CBS |
